# Co‐regulation of indole glucosinolates and camalexin biosynthesis by CPK5/CPK6 and MPK3/MPK6 signaling pathways

**DOI:** 10.1111/jipb.12973

**Published:** 2020-06-26

**Authors:** Liuyi Yang, Yan Zhang, Rongxia Guan, Sen Li, Xuwen Xu, Shuqun Zhang, Juan Xu

**Affiliations:** ^1^ State Key Laboratory of Plant Physiology and Biochemistry, College of Life Sciences Zhejiang University Hangzhou Zhejiang 310058 China; ^2^ Institute of Crop Sciences, Chinese Academy of Agricultural Sciences Beijing 100081 China; ^3^ Division of Biochemistry University of Missouri Columbia MO 65211 USA

**Keywords:** CPK5, MPK3/MPK6, camalexin, indole glucosinolate, immunity, *Botrytis cinerea*

## Abstract

Secondary plant metabolites, represented by indole glucosinolates (IGS) and camalexin, play important roles in *Arabidopsis* immunity. Previously, we demonstrated the importance of MPK3 and MPK6, two closely related MAPKs, in regulating *Botrytis cinerea* (*Bc*)‐induced IGS and camalexin biosynthesis. Here we report that CPK5 and CPK6, two redundant calcium‐dependent protein kinases (CPKs), are also involved in regulating the biosynthesis of these secondary metabolites. The loss‐of‐function of both *CPK5* and *CPK6* compromises plant resistance to *Bc*. Expression profiling of *CPK5‐VK* transgenic plants, in which a truncated constitutively active CPK5 is driven by a steroid‐inducible promoter, revealed that biosynthetic genes of both IGS and camalexin pathways are coordinately upregulated after the induction of CPK5‐VK, leading to high‐level accumulation of camalexin and 4‐methoxyindole‐3‐yl‐methylglucosinolate (4MI3G). Induction of camalexin and 4MI3G, as well as the genes in their biosynthesis pathways, is greatly compromised in *cpk5 cpk6* mutant in response to *Bc*. In a conditional *cpk5 cpk6 mpk3 mpk6* quadruple mutant, *Bc* resistance and induction of IGS and camalexin are further reduced in comparison to either *cpk5 cpk6* or conditional *mpk3 mpk6* double mutant, suggesting that both CPK5/CPK6 and MPK3/MPK6 signaling pathways contribute to promote the biosynthesis of 4MI3G and camalexin in defense against *Bc*.

## Graphical abstract

Plants produce secondary metabolites as defense chemicals in responses to biotic stimuli, including pathogens and herbivores. In this report, CPK5 and CPK6, two redundant calcium‐dependent protein kinases, are identified to play an important role in regulating the biosynthesis of indole glucosinolates and camalexin, represented antimicrobial metabolites in *Arabidopsis* immunity.

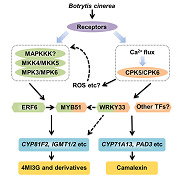

## INTRODUCTION

Plants produce secondary metabolites in response to biotic stimuli, including pathogens and herbivores (Kliebenstein 2004). These secondary metabolites play important roles in plant immunity (Bednarek 2012; Piasecka et al. 2015). Among them, glucosinolates (GSs) and camalexin have been extensively studied. Glucosinolates are a group of nitrogen‐ and sulfur‐rich functional defense‐related phytoanticipins and are derived from several amino acids (Halkier and Gershenzon 2006; Sonderby et al. 2010; Agerbirk and Olsen 2012). The biological activities of GSs are dependent on the release of various toxic hydrolytic products under the action of myrosinases (Agerbirk et al. 2008). According to their precursor amino acid and the types of modification to the R group, GSs can be classified as aliphatic glucosinolates (AGSs), aromatic glucosinolates (ARGSs), and indole glucosinolates (IGSs) (Piasecka et al. 2015). In *Arabidopsis*, the tryptophan‐derived IGSs are critical to plant immunity (Bednarek et al. 2009; Clay et al. 2009). Especially, 4‐methoxyindol‐3‐ylmethylglucosinolate (4MI3G) was found to be critical in defense against a variety of oomycetic and fungal pathogens (Schlaeppi and Mauch 2010; Schlaeppi et al. 2010; Stotz et al. 2011; Buxdorf et al. 2013). Camalexin is another important tryptophan‐derived indolic metabolite in response to a variety of pathogens and is the most prominent phytoalexin in *Arabidopsis* (Tsuji et al. 1992; Thomma et al. 1999; Stotz et al. 2011; Hiruma et al. 2013). Deficiency in camalexin production can lead to increased susceptibility of plants to several fungi, such as *Botrytis cinerea* and *Alternaria brassicicola* (Thomma et al. 1999; Ferrari et al. 2003; Ferrari et al. 2007).

Tryptophan is converted to indole‐3‐acetaldoxime (IAOx) in a reaction catalyzed by CYP79B2 and CYP79B3, two P450 enzymes (Zhao et al. 2002). IAOx can be converted to several indolic metabolites. When conjugation to GSH, a sulfur donor, and catalyzed by C‐S lyase SUR1, glucosyltransferases UGT74B1, and several sulfotransferases, IAOx converts to I3G (Mikkelsen et al. 2004; Sonderby et al. 2010). After hydroxylation and methylation by CYP81Fs and IGMT1/IGMT2, respectively, I3G finally converts to two main derivatives, 4MI3G and 1MI3G (Pfalz et al. 2011). In another branch, the cytochrome P450 enzymes CYP71A13, CYP71A12, and CYP71B15 (PAD3) catalyze camalexin synthesis from IAOx (Zhou et al. 1999; Schuhegger et al. 2006; Nafisi et al. 2007; Mucha et al. 2019). IAOx is also the key intermediate leading to the formation of a series of other low molecular weight indoles including the auxin indole‐3‐acetic acid (IAA) (Zhao et al. 2002).

Biosynthesis of IGSs and camalexin is tightly regulated, and several transcription factors have been identified to be involved. MYB34, MYB51, and MYB122 regulate IGS biosynthesis (Frerigmann and Gigolashvili 2014). Besides, MYC2, MYC3, and MYC4 are also implicated in regulating IGS levels by forming a complex with these MYBs (Schweizer et al. 2013). A recent study demonstrated that MPK3/MPK6 could phosphorylate its substrate ERF6, which promoted *B. cinerea*‐induced IGS biosynthesis and the conversion of I3G to 4MI3G by regulating the expression of *MYB51/MYB122* and *CYP81F2, IGMT1/IGMT2* (Xu et al. 2016). MPK3/MPK6, two redundant pathogen‐responsive mitogen‐activated protein kinases (MAPKs), are rapidly activated in response to pathogen infection and play critical roles in multiple plant defense responses (Rodriguez et al. 2010; Zhang et al. 2018). Previously, we also demonstrated the importance of MPK3/MPK6 upstream of WRKY33 in regulating camalexin biosynthesis in response to *Bc* (Ren et al. 2008; Mao et al. 2011). WRKY33 can directly bind to the promoter and upregulate the expression of *PAD3*, a gene that encodes the last enzyme in the camalexin biosynthetic pathway (Mao et al. 2011). In a recent ChIP‐Seq analysis, many other genes in the biosynthetic pathways of tryptophan‐derived indolic metabolites were found to be direct targets of WRKY33 (Birkenbihl et al. 2017).

Calcium‐dependent protein kinases (CPKs) are activated upon binding of Ca^2+^, converting the Ca^2+^ signal to a phosphorylation signal (Roberts and Harmon 1992; Harmon et al. 2000; Cheng et al. 2002; Romeis and Herde 2014). Calcium‐dependent protein kinases exhibit multiple functions in immune response, including oxidative burst, stomatal movements, hormonal signaling and gene regulation (Boudsocq and Sheen 2013). Among the gene family of 34 members encoding CPKs in *Arabidopsis*, CPK5 is of vital importance to plant immunity. In PTI, CPK5, together with CPK4, CPK6, and CPK11, plays an important role in flg22 signaling (Boudsocq et al. 2010). They also function redundantly in flg22‐induced ROS production and transcriptional reprogramming by directly mediating activity of transcription factors (Boudsocq et al. 2010; Dubiella et al. 2013). CPK5 phosphorylates the NADPH oxidase respiratory burst oxidase homolog D (RBOHD) in flg22‐triggered FLS2/BAK1‐BIK1/PBL1 signaling (Dubiella et al. 2013; Li et al. 2014). Moreover, CPK5 and its homologs are involved in positively mediating ETI and program cell death (PCD) by phosphorylating specific WRKYs, which are regulated by both CC‐ and TIR‐NB‐LRR protein (Gao et al. 2013; Peng et al. 2018). In addition, CPK5/CPK6 are involved in regulating ethylene biosynthesis in response to wounding or *B. cinerea* infection (Gravino et al. 2015; Li et al. 2018).

In this study, by using the conditional gain‐of‐function *GVG‐CPK5‐VK* and the loss‐of‐function *cpk5, cpk6*, and *cpk5 cpk6* single and double mutants, we demonstrated CPK5 and CPK6 function redundantly in promoting the accumulation of 4MI3G and camalexin in defense against *B. cinerea*. Both CPK5/CPK6 and MPK3/MPK6 signaling pathways could regulate secondary metabolites with antimicrobial activities and make important contribution to the resistance to *B. cinerea*.

## RESULTS

### *cpk5 cpk6* double mutant has compromised resistance to *B. cinerea*

**Figure 1:**
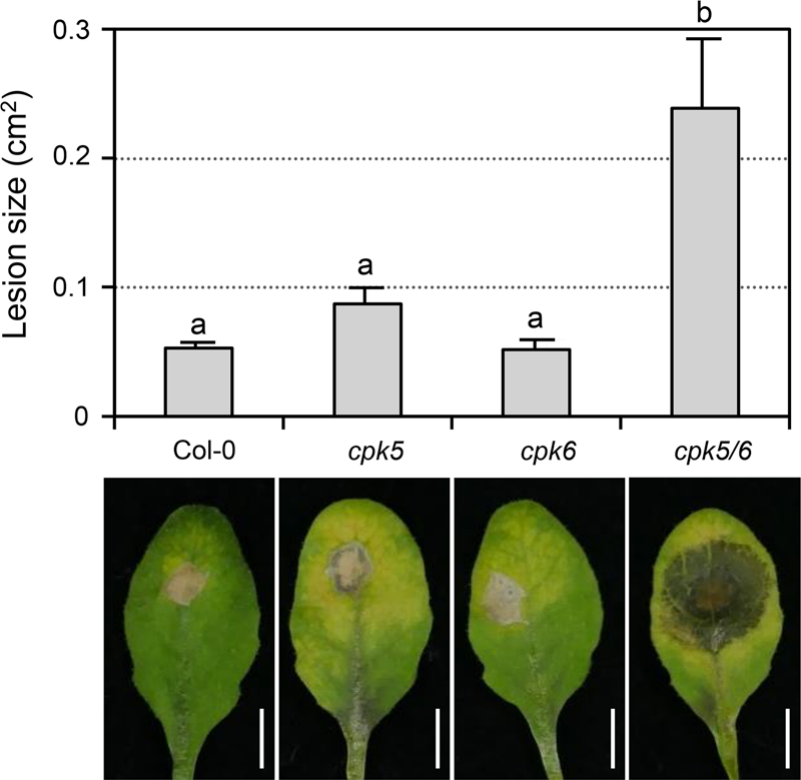
**Loss of function of both *CPK5* and *CPK6* compromises plant resistance to *B. cinerea*** Droplets of *B. cinerea* spore suspension (10 µL, 5 × 10^5^ spores/mL) were placed on detached leaves from 4‐week‐old soil‐grown Col‐0, *cpk5, cpk6*, and *cpk5 cpk6* plants. Photos were taken at 2.5 d after inoculation and lesion sizes were measured using ImageJ. Values are means ± SEM, *n* = 15–27. Spots represent every single value. One‐way ANOVA was performed to compare the lesion area of different genotypes (*P* < 0.05). Scale bar, 5 mm.

To assess the contribution of CPK5 in resistance to *B. cinerea*, we first quantified plant disease resistance of *cpk5* mutant. CPK5 belongs to clade I in the CPK gene family, in which CPK6 represents the closest homolog of CPK5 and plays redundant functions with CPK5 in flg22‐triggered immunity signaling (Harmon et al. 2001; Boudsocq et al. 2010). As a result, we checked the disease resistance of *cpk5, cpk6*, and *cpk5 cpk6* double mutants to *B. cinerea*. Newly fully developed leaves of 4‐week‐old Col‐0 and mutant plants were inoculated with droplets of *B. cinerea* spore suspension, and lesion size was measured 2.5 d after inoculation. No change in the susceptibility to *B. cinerea* was observed in either *cpk5* or *cpk6* single mutant in comparison to the wild type Col‐0 plants (Figure 1). However, lesions of *cpk5 cpk6* double mutant were significantly larger than that of wild‐type plants, indicating CPK5 and CPK6 play a redundant function in resistance against *B. cinerea*. This finding led us to search for the mechanism underlying CPK5/CPK6 in signaling plant immunity to *B. cinerea*.

### CPK5 activation up‐regulates genes in 4MI3G and camalexin biosynthetic pathways

It is reported that overexpression of CPK5 result in spontaneous cell death (Dubiella et al. 2013). To alleviate this problem and further investigate the role of CPK5 in plant immunity, we developed a conditional gain‐of‐function system *GVG‐CPK5‐VK* (abbreviated as *CPK5‐VK*). In this system, a constitutively active form of CPK5 was generated by deleting the C‐terminal Ca^2+^ regulatory and auto‐inhibitory domains, while retaining the N‐terminal variable domain and the kinase domain (Schulz et al. 2013). In this system, the truncated CPK5‐VK was expressed under the control of dexamethasone (DEX)‐inducible promoter. We treated the *CPK5‐VK* seedlings with DEX and did transcriptome profiling by RNA‐sequencing (abbreviated as RNA‐seq). The expression of 671 genes was changed significantly (Probability >0.9, fold change >2) in *CPK5‐VK* at 6 h after DEX treatment (Figure S1A).

**Figure 2:**
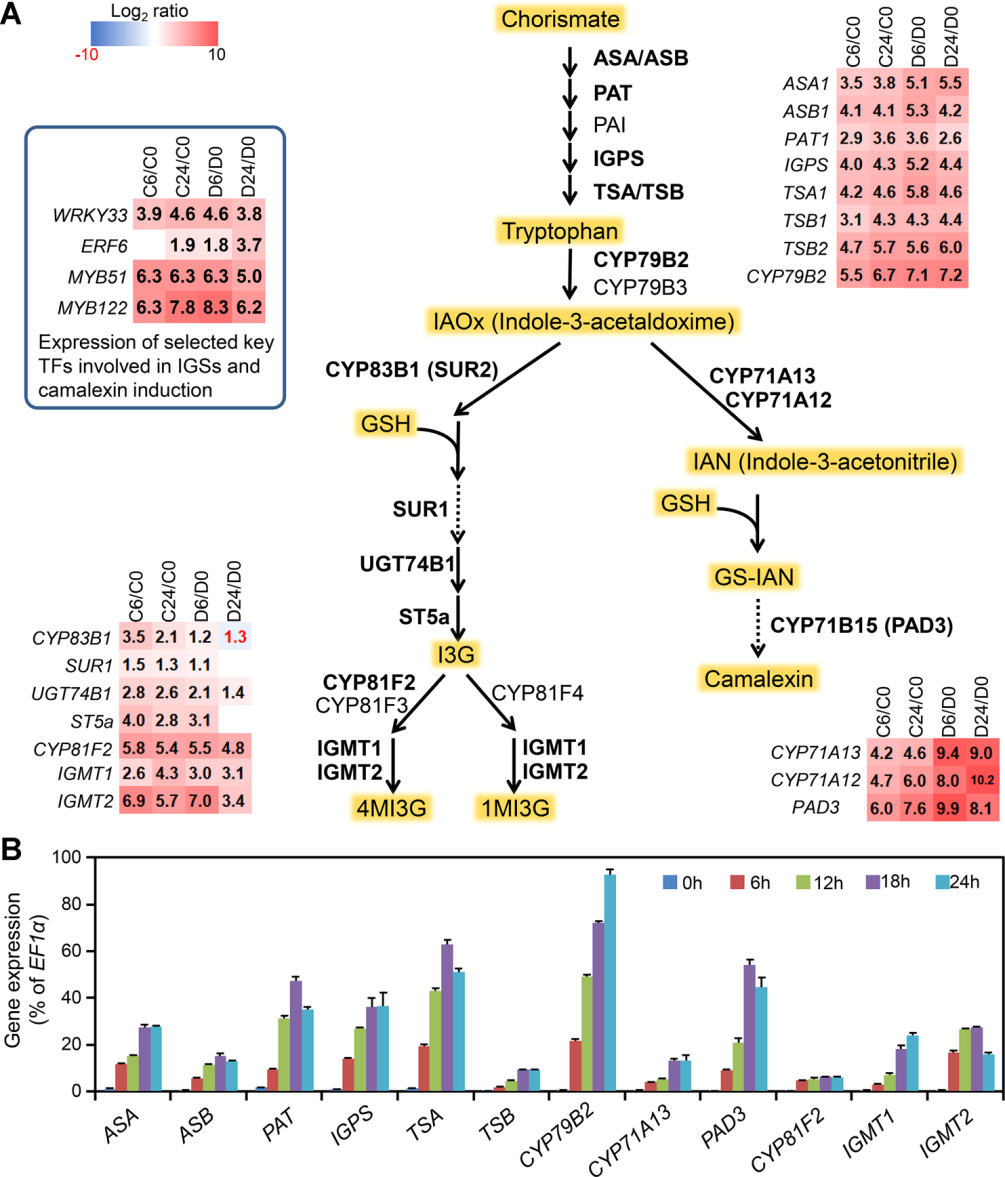
**Coordinated up‐regulation of genes in the IGS and camalexin biosynthetic pathways after gain‐of‐function activation of CPK5** (**A**) Schematic diagram of IGS and camalexin biosynthetic pathways. Changes of gene expression (in folds relative to 0 h samples, i.e., before treatment) are shown as a heatmap. C0, C6, and C24 are samples from *CPK5‐VK* plants before (0 h) or 6 h and 24 h after DEX treatment, respectively. D0, D6, and D24 are samples from *DD* plant before (0 h) or 6 h and 24 h after DEX treatment, respectively. Solid arrows indicate single enzymatic steps, whereas dashed lines stand for multiple enzymatic steps. Enzymes marked in bold font have elevated expression after CPK5 activation. (**B**) Activation of CPK5 induces up‐regulation of gens in IGS and camalexin biosynthesis pathway. Twelve‐d‐old *CPK5‐VK* plants grown in liquid medium were treated with 5 μM DEX for indicated times. Transcript levels were quantified by RT‐qPCR and calculated as percentages of the *EF1α* transcript. Values are means ± *SD, n* = 3.

Previously, we revealed the important roles of the MPK3/MPK6 cascade in resistance to *B. cinerea* (Ren et al. 2008). We also profiled gene expression changes after the gain‐of‐function activation of MPK3/MPK6 in *GVG‐NtMEK2* ^*DD*^ (abbreviated as *DD*) transgenic plants (Su et al. 2018). Comparative analysis revealed that approximately 84% (562 in 671) of the differentially expressed genes (DEGs) in *CPK5‐VK* plants were shared in *DD* plants (Figure S1A). Among these 562 DEGs, 333 genes were up‐regulated and 229 genes were down‐regulated in both plants (Figure S1A and B, Data Set S1). GO analysis of the 333 up‐regulated genes revealed a significant enrichment in genes involved in the metabolism of indole‐ and sulfur‐containing compounds (Figure S1C). Next, we examined the expression changes of the genes encoding known enzymes in the biosynthetic pathways of IGS and camalexin, two important tryptophan derived compounds in plant defense. As shown in Figure 2A, the expression of most genes in the biosynthesis pathways of IGS and camalexin was up‐regulated after the gain‐of‐activation of both CPK5 and MPK3/MPK6. RT‐qPCR analysis further confirmed the up‐regulation of these genes after the activation of CPK5 (Figure 2B). These results indicated that the activation of CPK5 might be able to induce the biosynthesis of IGS and camalexin, two important antimicrobial compounds in *Arabidopsis* immunity.

### 4MI3G and camalexin both accumulate after CPK5 activation

**Figure 3:**
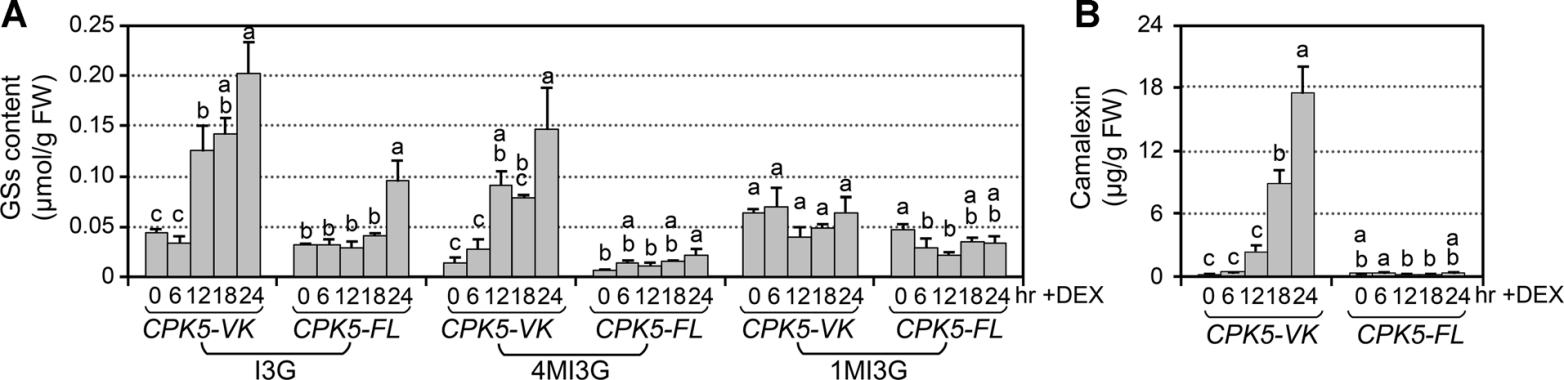
**Activation of CPK5 induces accumulation of 4MI3G and camalexin** Twelve‐d‐old *CPK5‐VK* and *CPK5‐FL* plants grown in liquid medium were treated with 5 μM DEX for indicated times. (**A**) Glucosinolates were measured using HPLC. (**B**) Levels of camalexin in the medium were measured using a microtiter plate reader at indicated time points. Values are means ± *SD, n* = 3. One‐way ANOVA was performed to compare the levels of GSs and camalexin at different time point after treatment. Lowercase letters above the columns indicate statistically different time points (*P* < 0.05). FW: Fresh weight.

To find out whether CPK5 activation is sufficient to induce IGS and camalexin biosynthesis, cellular levels of three main indole‐GSs (I3G, 4MI3G, and 1‐methoxy‐3‐indolylmethyl‐GS (1MI3G)) and secreted camalexin in the medium after CPK5 activation in *CPK5‐VK* plants were measured. In this experiment, we used *GVG‐CPK5‐FL* transgenic plants (*CPK5‐FL* for short), in which a full‐length *CPK5* transgene is driven by the steroid‐inducible promoter, as a negative control. Induction of *CPK5‐FL* expression after DEX treatment is not sufficient to trigger downstream response because the full‐length CPK5 is inhibited by its auto‐inhibitory domain and requires an elevated calcium level in response to an external stimulus for activation. As shown in Figure 3, in the *CPK5‐VK* seedlings, DEX treatment greatly induced the accumulation of I3G, 4MI3G, and camalexin, which is consistent with the RNA‐Seq results. The levels of I3G, 4MI3G, and camalexin increased about 4, 11, and 84 folds, respectively, in *CPK5‐VK* seedlings after 24 h of DEX treatment. Whereas in the *CPK5‐FL* seedlings, the increase in I3G, 4MI3G, or camalexin was much lower than that in *CPK5‐VK*. The level of 1MI3G has no significant change in *CPK5‐VK* and slightly decreased in *CPK5‐FL* seedlings (Figure 3A). Taken together, activation of CPK5 could drive the metabolism to the biosynthesis of I3G and its conversion to 4MI3G, as well as the biosynthesis of camalexin.

### **CPK5 and CPK6 function redundantly in promoting 4MI3G or camalexin accumulation in response to** *B. cinerea* **infection**

**Figure 4:**
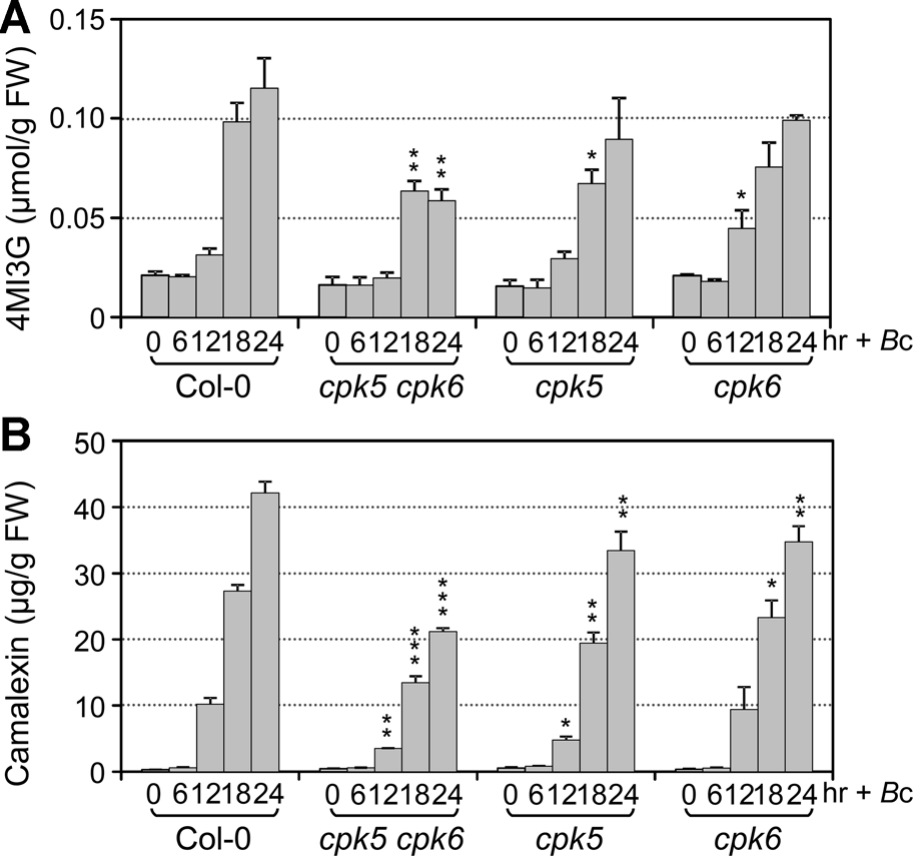
***B. cinerea*‐induced 4MI3G and camalexin biosynthesis is compromised in *cpk5 cpk6* double mutant** Twelve‐d‐old Col‐0, *cpk5 cpk6, cpk5*, and *cpk6* plants grown in liquid medium were treated with *B. cinerea* spores (4 × 10^5^ spores/mL). Levels of 4MI3G (**A**) and camalexin (**B**) were measured at indicated time points. Values are means ± *SD, n* = 3. One‐way ANOVA was performed to compare the levels of 4MI3G or camalexin in mutants and Col‐0 at 12 h, 18 h, and 24 h, *P < 0.05, **P < 0.01, ***P < 0.001.

**Figure 5:**
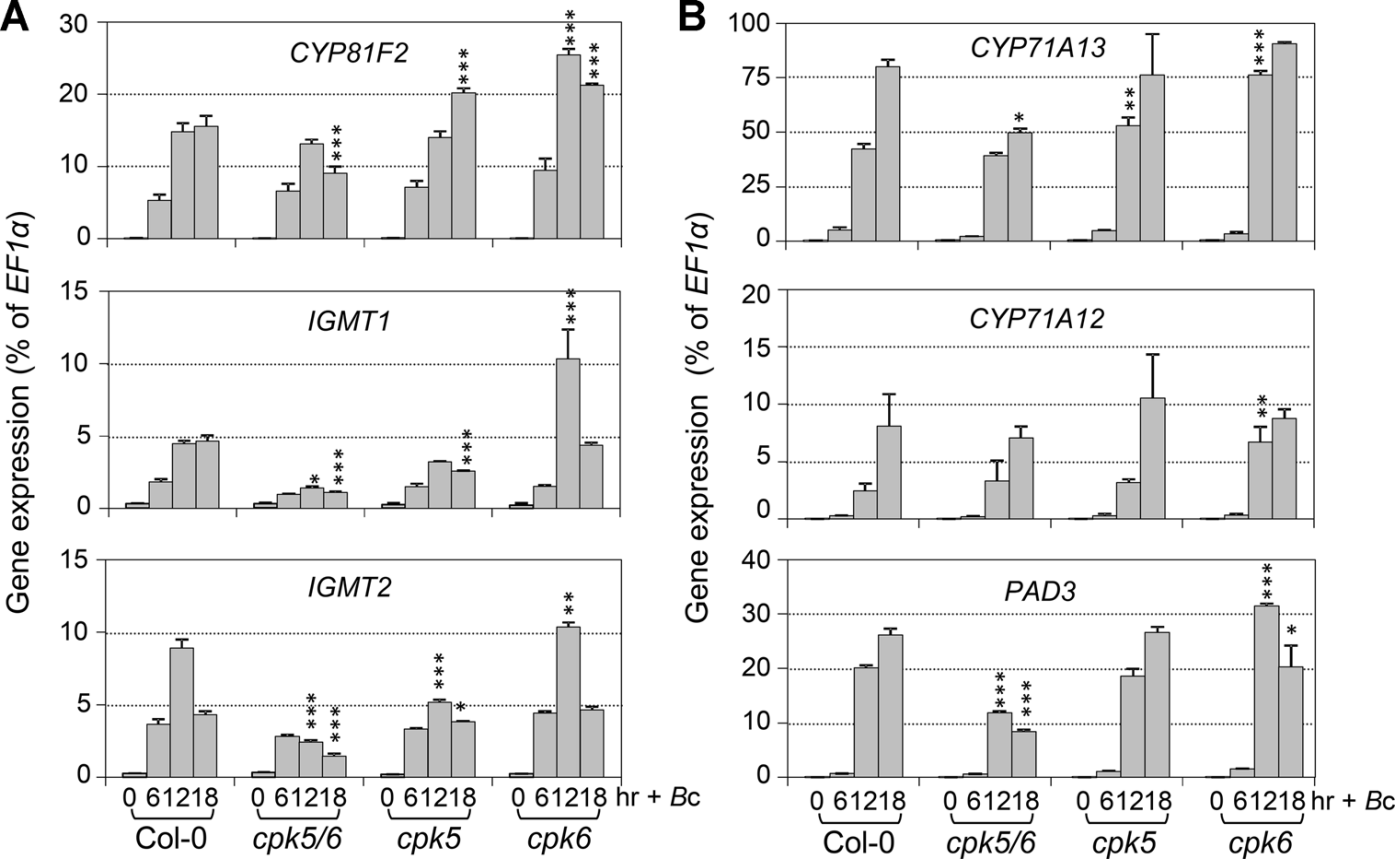
***B. cinerea*‐induced activation of 4MI3G and camalexin biosynthetic genes is compromised in *cpk5 cpk6* double mutant** Twelve‐d‐old Col‐0, *cpk5 cpk6, cpk5* and *cpk6* plants grown in liquid medium were treated with *B. cinerea* spores (4 × 10^5^ spores/mL) for indicated times. Expression levels of 4MI3G (**A**) and camalexin (**B**) biosynthetic genes were determined by RT‐qPCR and calculated as percentages of the *EF1α* transcript. Values are means ± *SD, n* = 3. One‐way ANOVA was performed to compare gene expression level of each mutant to that of Col‐0 at 12 h and 18 h, *P < 0.05, **P < 0.01, ***P < 0.001.

To provide loss‐of‐function evidence to support the role of CPK5 in the induction of 4MI3G and camalexin, we measured cellular levels of I3G, 4MI3G, and secreted camalexin in *cpk5* and *cpk6* single, and *cpk5 cpk6* double mutants after *B. cinerea* inoculation. As shown in Figure 4, the levels of *B. cinerea*‐induced 4MI3G in *cpk5* or *cpk6* single mutant were comparable to that in Col‐0, and the levels of camalexin in these two single mutants reduced slightly. But in *cpk5 cpk6* double mutant, *B. cinerea*‐induced 4MI3G and camalexin reduced by approximately 50% (Figure 4). Consistent with this, RT‐qPCR analysis revealed that the up‐regulation of key biosynthetic genes of 4MI3G and camalexin was significantly compromised in *cpk5 cpk6* mutant in comparison to that in Col‐0 after inoculation with *B. cinerea* (Figure 5). For instance, genes encoding the enzymes that catalyze the conversion of I3G to 4MI3G, including *CYP81F2, IGMT1*, and *IGMT2*, and that catalyze the key steps of camalexin biosynthesis, including *CYP71A13* and *PAD3*. Meanwhile, the expression of these genes in *cpk5* and *cpk6* single mutant showed no decrease or relatively small change. Taken together, these results showed that CPK5 and CPK6 function redundantly in driving the synthesis of 4MI3G and camalexin by up‐regulating the expression of genes in their biosynthetic pathways after *B. cinerea* infection.

In addition, the expression of *ASA, ASB, PAT, IGPS, TSA, TSB*, and *CYP79B2*, the genes that encode enzymes catalyzing the conversion of Chorismate to IAOx (Indole‐3‐acetaldoxime), was all reduced in *cpk5 cpk6* double mutant 18 h after *B. cinerea* inoculation (Figure S2). Interestingly, despite the reduction in gene expression in IAOx biosynthesis pathway, we found that the cellular level of *B. cinerea*‐induced I3G showed no change in *cpk5 cpk6* double mutant in comparison to Col‐0 (Figure S3). The I3G level could be an integrated result of the expression of genes in the whole metabolism pathway. We noticed that the induction of *CYP83B1*, the gene encoding the first enzyme that catalyzes the conversion from IAOx to I3G was much higher in *cpk5 cpk6* plants, and expression of *UGT74B1* and *ST5a* only slightly reduced in the double mutant (Figure S4), which might be one reason for *B. cinerea*‐induced accumulation of I3G wasn't reduced in the *cpk5 cpk6* double mutant (Figure S3). On the other hand, the compromised expression of *CYP81F2* also reduced the consumption of I3G.

### *MYB51* **is downstream of CPK5/CPK6 in** *B. cinerea* **‐induced IGS biosynthesis**

**Figure 6:**
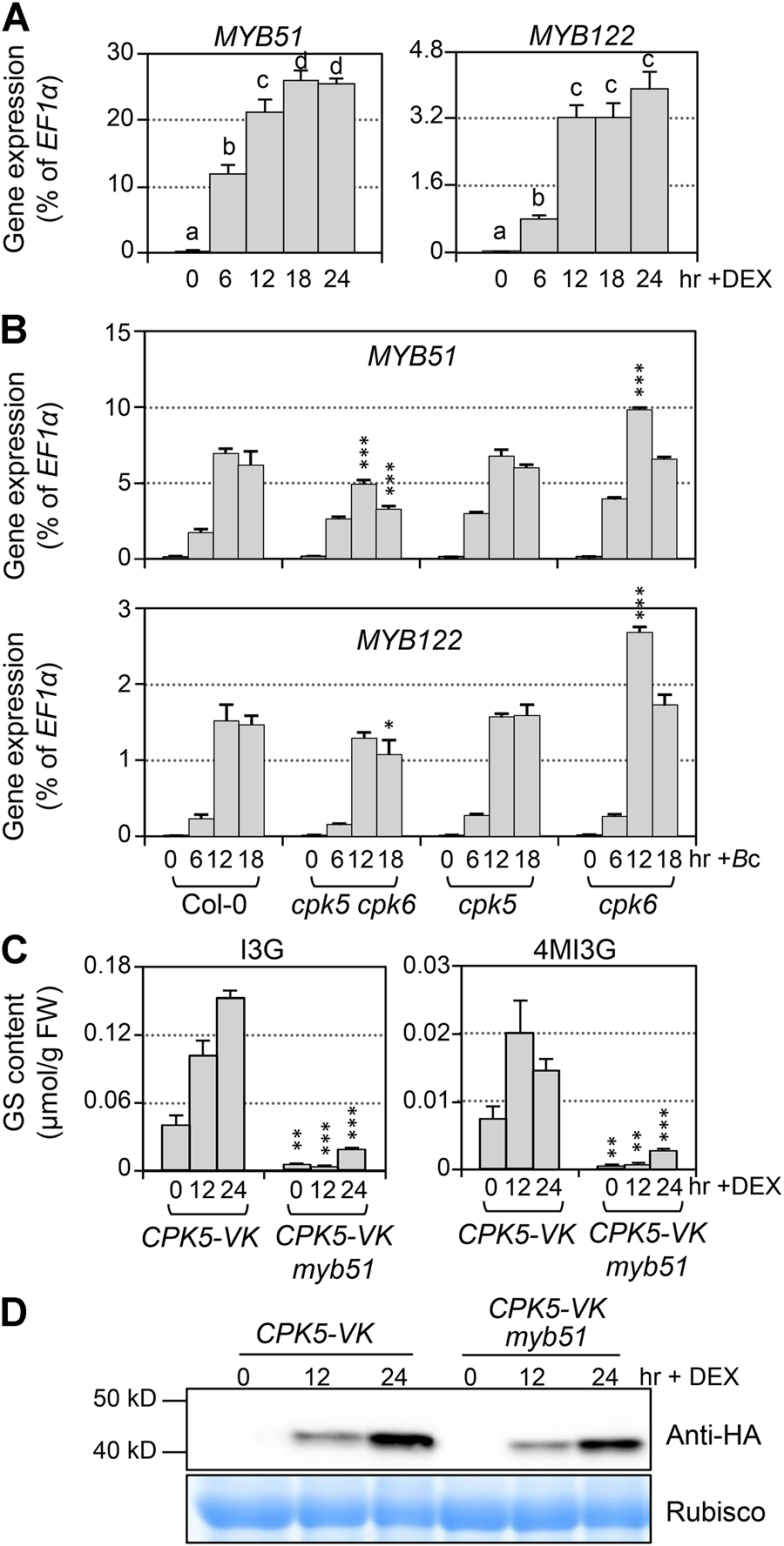
**MYB51 is downstream of CPK5/CPK6 in *B. cinerea*‐induced IGS biosynthesis** (**A**) Twelve‐d‐old *CPK5‐VK* plants grown in liquid medium were treated with 5 μM DEX for indicated times. Transcript levels were quantified by RT‐qPCR and calculated as percentages of the *EF1α* transcript. Values are means ± *SD, n* = 3. One‐way ANOVA was performed to compare gene expression level at different time point after treatment. Lowercase letters above the columns indicate statistically different time points (*P* < 0.05). (**B**) Twelve‐d‐old Col‐0, *cpk5 cpk6, cpk5*, and *cpk6* plants grown in liquid medium were treated with *B. cinerea* spores (4×10^5^ spores/mL) for indicated times. Transcript levels were quantified by RT‐qPCR and calculated as percentages of the *EF1α* transcript. Values are means ± *SD, n* = 3. One‐way ANOVA was performed to compare the levels of each gene expression between each mutant and Col‐0 at 12 h and 18 h, *P < 0.05, ***P < 0.001. (**C**) Twelve‐d‐old *CPK5‐VK* and *CPK5‐VK myb51* plants grown in liquid medium were treated with 5 μM DEX. Levels of I3G and 4MI3G were measured at indicated time points. Values are means ± *SD, n* = 3. Student *t*‐test was performed to compare the levels of I3G or 4MI3G between *CPK5‐VK* and *CPK5‐VK myb51* at each time point, **P < 0.01, ***P < 0.001. (**D**) CPK5‐VK protein in WT and *myb51* background was detected by western blot to show the same induction. CBB (Coomassie brilliant blue) staining was used to show equal loading.

MYB51/MYB122/MYB34 transcription factors play indispensable roles in regulating IGS biosynthesis (Gigolashvili et al. 2007; Frerigmann and Gigolashvili 2014). It was reported that MYB34 controls the biosynthesis of IGSs mainly in the roots, MYB51 regulates IGS biosynthesis in shoots, and MYB122 has an accessory role (Frerigmann and Gigolashvili 2014). RNA‐Seq results showed that *MYB51* and *MYB122* were highly up‐regulated after CPK5 activation (Figure 2A). Consistent with this, RT‐qPCR results also showed the high induction of *MYB51* and *MYB122* after CPK5 activation (Figure 6A). We then analyzed expression levels of *MYB51* and *MYB122* in the mutant plants after *B. cinerea* inoculation. The expression of *MYB51* was significantly compromised in the *cpk5 cpk6* double mutant, while *MYB122* expression was only slightly reduced (Figure 6B). To further determine whether *MYB51* functions downstream of CPK5, we crossed *CPK5‐VK* into *myb51* mutant plants, and detected the induction of IGSs after DEX treatment. As shown in Figure 6C, both I3G and 4MI3G decreased dramatically in *CPK5‐VK myb51* plants. These results suggest that CPK5, redundantly with CPK6, regulates I3G biosynthesis through *MYB51* in response to *B. cinerea*.

### **CPK5/CPK6 and MPK3/MPK6 function collaboratively in** *B. cinerea* **‐induced 4MI3G and camalexin**

**Figure 7:**
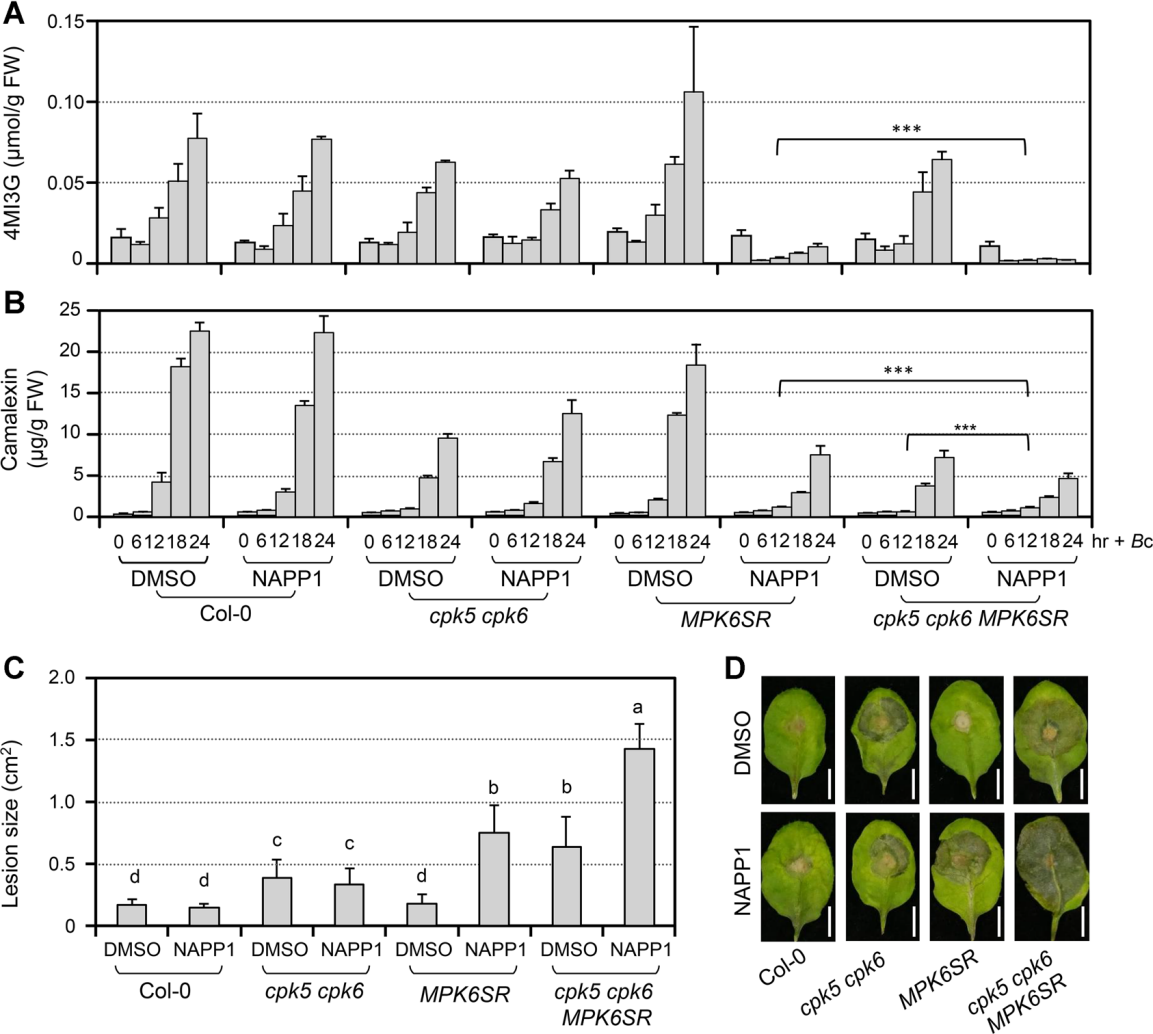
**Loss‐of‐function of both *CPK5/CPK6* and *MPK3/MPK6* further reduces 4MI3G and camalexin induction and resistance in defense to *B. cinerea*** (**A**,**B**) Loss‐of‐function of both CPK5/CPK6 and MPK3/MPK6 resulted in reduced 4MI3G or camalexin accumulation in comparison to that in *cpk5 cpk6* or *mpk3 mpk6* double mutant after *B.cinerea* inoculation. Col‐0, *cpk5 cpk6, MPK6SR*, and *cpk5 cpk6 MPK6SR* seedlings were pretreated with DMSO (solvent of NA‐PP1stock) or NA‐PP1 (final concentration of 2.5 μM) for 30 min before *B. cinerea* (4 × 10^5^ spores/mL) inoculation. Seedlings and medium were collected at indicated times for GS assay (**A**) and camalexin measurement (**B**) respectively. Values are means ± *SD, n* = 3. Group differences were analyzed by two‐way ANOVA, ***P < 0.001. (**C**,**D**) Loss‐of‐function of both CPK5/CPK6 and MPK3/MPK6 resulted in compromised resistance to *B. cinerea* in comparison to *cpk5 cpk6* or *mpk3 mpk6* double mutant. Detached leaves from 4‐week‐old soil‐grown *cpk5 cpk6, MPK6SR*, and *cpk5 cpk6 MPK6SR* plants were put on wet filter paper with 10 μM NA‐PP1 or DMSO (as solvent control) and inoculated with 10 μL droplets of a suspension of *B. cinerea* spores (5 × 10^5^ spores/mL). Photos were taken after 2.5 d (**D**) and lesion sizes (**C**) were measured by ImageJ. Values are means ± *SD, n* = 20–24. One‐way ANOVA was performed to compare the lesion area of different genotypes and treatments (*P* < 0.05). Scale bar, 5 mm.

In response to pathogen infection, both CPK and MAPK signaling pathways can be rapidly activated (Hake and Romeis 2019). Our previous reports and this study reveal that both CPK5/CPK6 and MPK3/MPK6 are involved in regulating *B. cinerea*‐induced 4MI3G and camalexin (Ren et al. 2008; Xu et al. 2016; He et al. 2019). What is the relationship between these two pathways in this process? To answer this question, we constructed a loss‐of‐function of CPK5/CPK6 and MPK3/MPK6 system. Because *mpk3 mpk6* mutant is lethal, we crossed *cpk5 cpk6* double mutant to *MPK6SR*, a chemical genetically rescued *mpk3 mpk6* mutant sensitized to NA‐PP1 (Xu et al. 2014). When treated with NA‐PP1, the *cpk5 cpk6 MPK6SR* plant is equal to *cpk5 cpk6 mpk3 mpk6* quadruple mutant. As shown in Figure 7A, B, the *B. cinerea*‐induced accumulation of 4MI3G and camalexin were further compromised in *cpk5 cpk6 MPK6SR* seedlings in comparison to *MPK6SR* or *cpk5 cpk6* double mutant under NA‐PP1 pretreatment condition. This result indicated that CPK5/CPK6 and MPK3/MPK6 could work collaboratively in regulating *B. cinerea*‐induced accumulation of 4MI3G and camalexin. Furthermore, consistent with the reduction in 4MI3G and camalexin, the NA‐PP1‐treated quadruple mutant showed significantly enhanced susceptibility to *B. cinerea* (Figure 7C, D).

To further understand the relationship between CPK5/CPK6 and MPK3/MPK6 in signaling 4MI3G and camalexin biosynthesis, we tried to cross *CPK5‐VK* to *MPK6SR* for determining whether the induction of 4MI3G or camalexin after CPK5 activation is dependent on MPK3/MPK6. Unfortunately, because of the genetic complexity of the *MPK6SR* background (two T‐DNA insertional mutations and one transgene), CPK5‐VK was silenced in the crossed plants. This led us to the available *mkk4 mkk5* TILLING mutant (Zhao et al. 2014; Su et al. 2017), which might provide an alternative solution to replace the silenced *CPK5‐VK MPK6SR* material.

**Figure 8:**
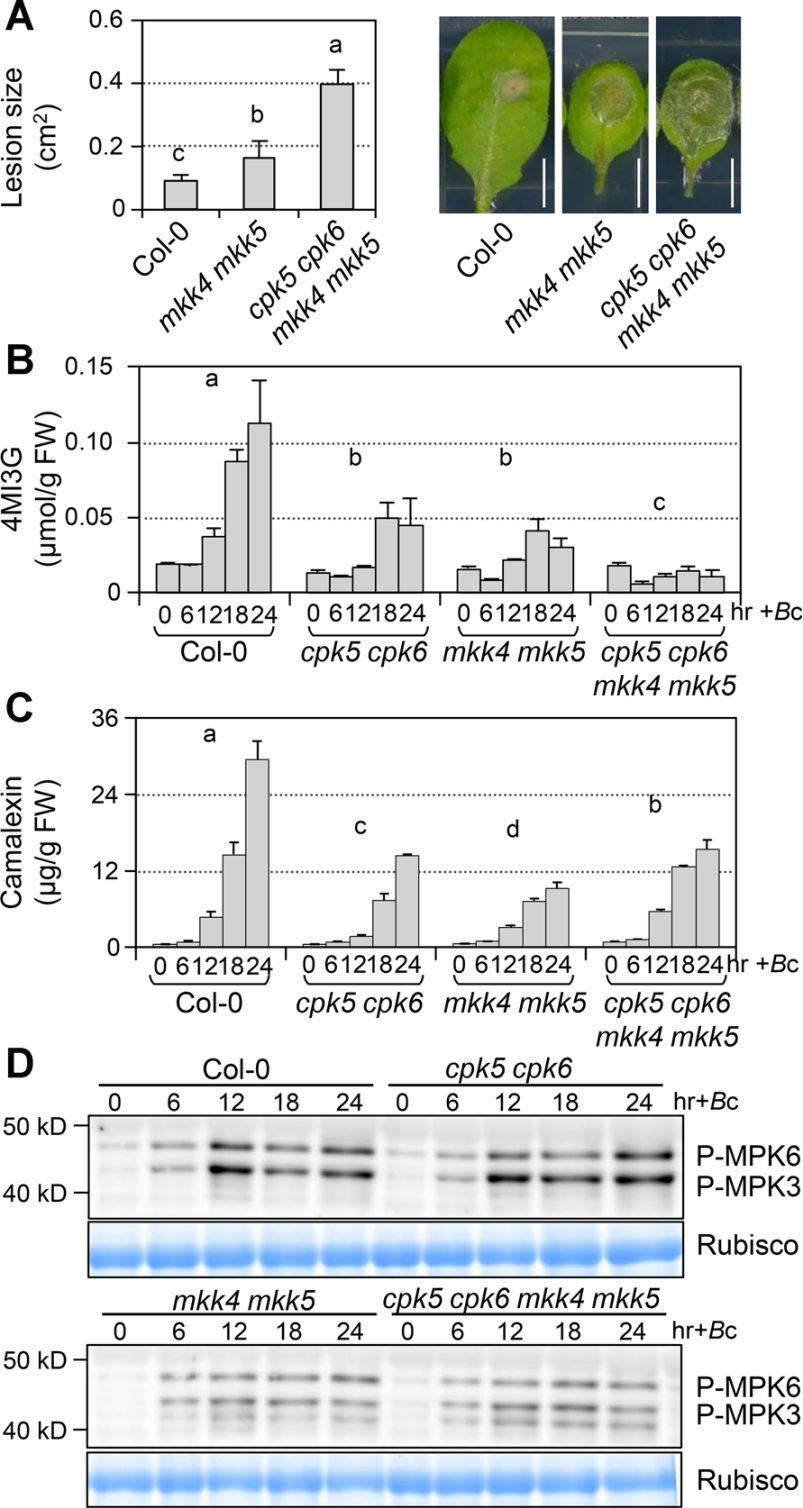
**MKK4/MKK5 are upstream of MPK3/MPK6 in regulating** ***B. cinerea*** **‐induced 4MI3G and camalexin** (**A**) *mkk4 mkk5* double mutant and *cpk5 cpk6 mkk4 mkk5* quadruple mutant show compromised resistance to *B. cinerea*. Droplets of *B. cinerea* spore suspension (10 μL, 5 × 10^5^ spores/mL) were placed on detached leaves from 4‐week‐old soil‐grown Col‐0, *mkk4 mkk5*, and *cpk5 cpk6 mkk4 mkk5* plants. Photos were taken after 2.5 d and lesion sizes were measured by ImageJ. Values are means ± *SD, n* = 15. One‐way ANOVA was performed to compare the lesion size of different genotypes (*P* < 0.001). Scale bar, 5 mm. (**B**,**C**) Twelve‐d‐old Col‐0, *cpk5 cpk6, mkk4 mkk5*, and *cpk5 cpk6 mkk4 mkk5* plants grown in liquid medium were treated with *B. cinerea* spores (4 × 10^5^ spores/mL). Levels of 4MI3G and camalexin were measured at indicated time points. Values are means ± *SD, n* = 3. Group differences were analyzed by two‐way ANOVA (*P* < 0.001). (**D**) Activation of MPK3/MPK6 in response to *B. cinerea* was compromised in *mkk4 mkk5* mutant. Activation of MPK6 and MPK3 was determined by immunoblot analysis using anti‐pTEpY antibody. Equal loading of proteins was confirmed by Coomassie brilliant blue staining.

MKK4 and MKK5 were reported to be redundant MAPKKs upstream of MPK3/MPK6 in signaling multiple developmental processes (Xu and Zhang 2015) and defense responses (Su et al. 2017; Li et al. 2018). Whether MKK4/MKK5 function upstream of MPK3/MPK6 in *B. cinerea*‐induced accumulation of 4MI3G or camalexin is still unknown. We first quantified the resistance to *B. cinerea* of *mkk4 mkk5* double mutant and detected the accumulation of 4MI3G and camalexin after *B. cinerea* inoculation. The *mkk4 mkk5* double mutant showed compromised resistance to *B. cinerea* (Figure 8A). Similar to the *mpk3 mpk6* mutant (Xu et al. 2016), the *B. cinerea*‐induced 4MI3G and camalexin is greatly decreased in *mkk4 mkk5* mutant as well (Figure 8B, C). In addition, activation of MPK3/MPK6 is greatly compromised in *mkk4 mkk5* double mutant after *B. cinerea* inoculation (Figure 8D). The residual activation of MPK3 and MPK6 in *mkk4 mkk5* double mutant could result from the approximately 10% residual activity of MKK4 in the *mkk4 mkk5* TILLING mutant. These results indicated that MKK4/MKK5 function upstream of MPK3/MPK6 in regulating 4MI3G and camalexin biosynthesis and resistance in response to *B. cinerea*. Meanwhile, we also generated and analyzed the *cpk5 cpk6 mkk4 mkk5* quadruple mutant, which showed significantly enhanced susceptibility to *B. cinerea* and reduced induction of 4MI3G compared to *cpk5 cpk6* or *mkk4 mkk5* double mutant (Figure 8A–C). However, *B. cinerea*‐induced camalexin in the quadruple mutant showed no further decrease compared to both double mutants. In addition, we observed that the phosphorylation activation of MPK3 and MPK6 in *cpk5 cpk6* double and *cpk5 cpk6 mkk4 mkk5* quadruple mutant is comparable to that in Col‐0 and *mkk4 mkk5*, respectively, after *B. cinerea* inoculation (Figure 8D), suggesting that loss of function of CPK5/CPK6 pathway has no effect on the activation of MPK3/MPK6 signaling pathway. Collectively, these results indicated that both MKK4/MKK5‐MPK3/MPK6 and CPK5/CPK6 signaling pathways contribute to promote the 4MI3G and camalexin biosynthesis in resistance to *B. cinerea*.

### Activation of MPK3/MPK6‐ or CPK5‐induced accumulation of 4MI3G and camalexin is independent to each other

**Figure 9:**
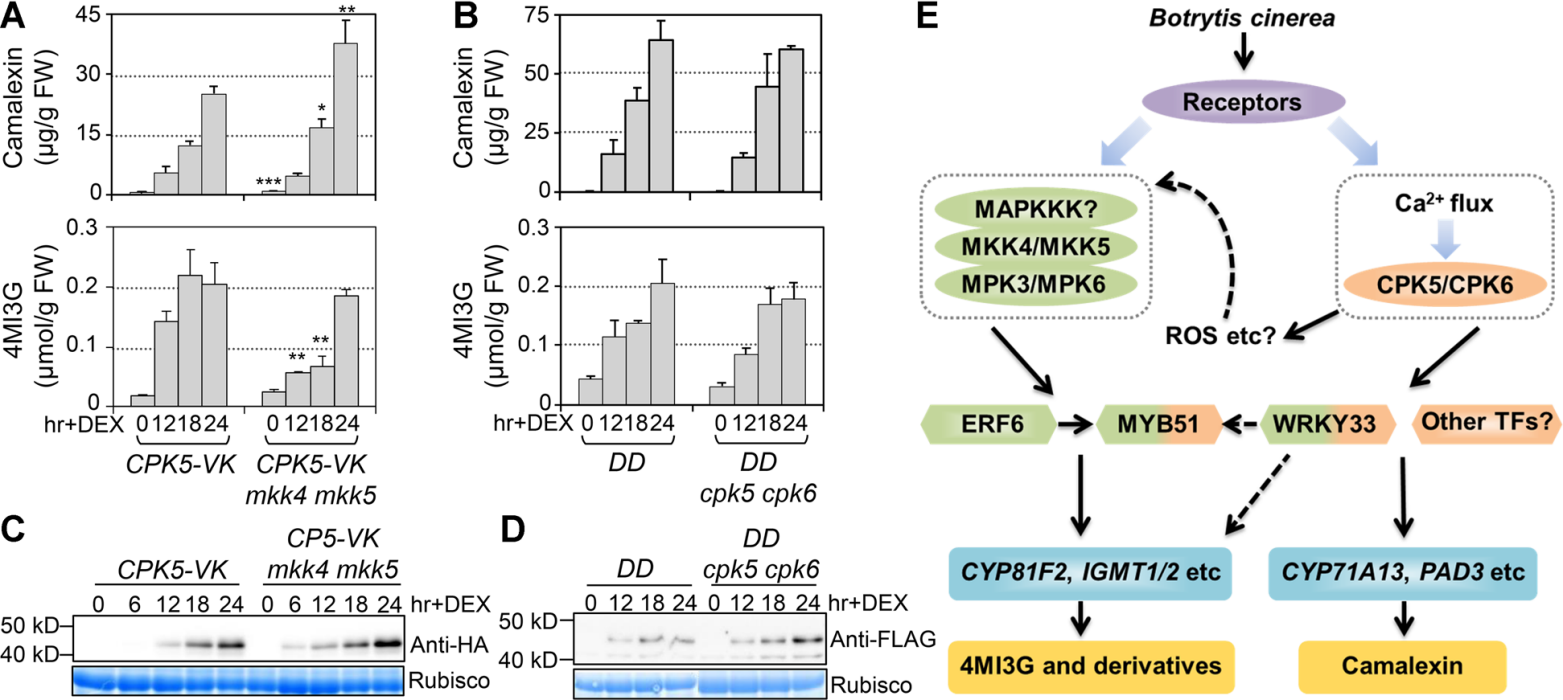
**Independent functions of CPK5 and MPK3/MPK6 signaling in 4MI3G and camalexin induction** (**A**) Twelve‐d‐old *CPK5‐VK* and *CPK5‐VK mkk4 mkk5* plants grown in liquid medium were treated with 5 μM DEX. Student *t*‐test was performed to compare the levels of camalexin or 4MI3G between *CPK5‐VK mkk4 mkk5* and *CPK5‐VK* at each time point, *P < 0.05, **P < 0.01, ***P < 0.001. (**B**) *DD* and *DD cpk5 cpk6* plants grown in liquid medium were treated with 5 μM DEX. Camalexin and 4MI3G were measured at indicated time points. Two‐way ANOVA analysis revealed that no significant group difference exists in camalexin and 4MI3G level change between different genotypes. Values are means ± *SD, n* = 3. FW: Fresh weight. (**C**) Protein levels of CPK5‐VK in *CPK5‐VK* and *CPK5‐VK mkk4 mkk5* plants after DEX treatment were determined by immunoblot analysis using an anti‐HA antibody. (**D**) Protein levels of NtMEK2^DD^ in *DD* and *DD cpk5 cpk6* plants after DEX treatment were determined by immunoblot analysis using an anti‐FLAG antibody. Equal loading of proteins was confirmed by Coomassie brilliant blue staining. (**E**) A model depicts the independent and cooperative interaction of MPK3/MPK6 cascade and CPK5/CPK6 signaling in regulating 4MI3G and camalexin biosynthesis in response to *B. cinerea* infection. In response to *B. cinerea* infection, both CPK5/CPK6 and MPK3/MPK6 signaling pathways can be rapidly activated. The secondary responses/signals, such as ROS, after activation of CPK5/CPK6 could result in the change of the activity of MPK3/MPK6. The interplay between CPK5/CPK6 and MPK3/MPK6 also could converge on their common downstream targets, including the transcription factors WRKY33 and MYB51, to fine‐tune the downstream defense response.

Since MKK4 and MKK5 are upstream of MPK3 and MPK6 in regulating *B. cinerea*‐induced 4MI3G and camalexin biosynthesis, we crossed CPK5‐VK plants to *mkk4 mkk5* mutant and generated *CPK5‐VK mkk4 mkk5* plants. We found that CPK5 activation‐induced camalexin accumulation was not compromised but even slightly higher in the *mkk4 mkk5* double mutant (Figure 9A). In contrast, the CPK5 activation‐induced accumulation of 4MI3G was delayed in the *mkk4 mkk5* background. However, the accumulation of 4MI3G caught up at later timepoints in the *mkk4 mkk5* background (Figure 9A). Comparable CPK5‐VK expression levels in Col‐0 and *mkk4 mkk5* mutant background were confirmed by western‐blot analysis (Figure 9C).

On the other hand, to determine whether MPK3/MPK6 activation‐induced biosynthesis of 4MI3G and camalexin was dependent on CPK5/CPK6, we introduced *GVG‐NtMEK2* ^*DD*^ (abbreviated as *DD*) transgene into *cpk5 cpk6* double mutant background by crossing. As we reported before, activation of MPK3 and MPK6 in *DD* plants would highly induce biosynthesis of 4MI3G and camalexin (Ren et al. 2008; Xu et al. 2016). Neither induction of 4MI3G nor camalexin accumulation in *DD* plants was affected by loss‐of‐function of CPK5/CPK6 (Figure 9B, D), suggesting that induction of 4M3G and camalexin by activation of MPK3/MPK6 is independent of CPK5/CPK6. These results indicated that gain‐of‐function activation of CPK5/CPK6 and MKK4/MKK5‐MPK3/MPK6 could independently regulate 4MI3G and camalexin biosynthesis.

## DISCUSSION

The Trp‐derived metabolites, represented by IGSs and camalexin, are powerful antimicrobial chemicals in plant resistance against pathogens (Bednarek 2012; Piasecka et al. 2015). Key enzymes/genes in the IGSs and camalexin biosynthetic pathways, as well as transcription factors upstream, have been identified (Ren et al. 2008; Frerigmann and Gigolashvili 2014; Xu et al. 2016; Mucha et al. 2019). However, the signaling pathways involved in the regulation of these transcription factors remain largely unknown. Previously, we reported that MPK3/MPK6 cascade plays an important role in regulating IGSs and camalexin biosynthesis in resistance to *B. cinerea* (Ren et al. 2008; Mao et al. 2011; Xu et al. 2016). In this report, we identified the important role of another signaling pathway, CPK5/CPK6, in regulating the biosynthesis of IGSs and camalexin in response to *B. cinerea*. In searching for the underlying mechanism of the compromised resistance in the *cpk5 cpk6* mutant (Figure 1), we found that CPK5/CPK6 are essential for the full induction of 4MI3G and camalexin in response to *B. cinerea* infection (Figures 4 and 5). Loss‐of‐function of both CPK5/CPK6 and MPK3/MPK6 pathways resulted in a significant lower *B. cinerea*‐induced of 4MI3G and camalexin and enhanced susceptibility of plants to *B. cinerea* than loss‐of‐function of either pathway (Figure 7). Our genetic analyses suggest that CPK5/CPK6 and MPK3/MPK6 signaling pathway could collaboratively, yet independently, regulate the biosynthesis of these important secondary metabolites and plant resistance in response to *B. cinerea* infection.

Calcium signals play a fundamental role in plant defense responses. Among the CPKs, the role of CPK5 in plant immunity has been extensively studied in recent years. In response to bacterial pathogens, CPK5, together with CPK4, CPK6, and CPK11, was implicated in both PTI and ETI (Boudsocq et al. 2010). CPK5 also plays a unique role in resistance to the powdery mildew pathogen *Golovinomyces cichoracearum*, a biotrophic fungus pathogen, by directly interacting with the atypical immune receptor TN2, a truncated leucine‐rich repeat (NLR) protein (Liu et al. 2017). The interaction with TN2 is able to stabilize and activate CPK5 to regulating immune responses, including cell death, expression of defense genes, and SA accumulation. However, the role of CPK5 in resistance to necrotrophic fungus *B. cinerea* is largely unknown. Previous research showed CPK5/CPK6 function in *B. cinerea*‐induced plant immunity by regulating ethylene biosynthesis (Gravino et al. 2015). The study of the function of CPK5 was limited because CPK5‐VK overexpression displayed spontaneous cell death (Dubiella et al. 2013). To solve this problem, we developed a conditional gain‐of‐function system *CPK5‐VK*. RNA‐seq data of *CPK5‐VK* provided an important clue that CPK5 is involved in regulating the biosynthesis of secondary metabolites with antimicrobial activities (Figure S1 and S2). Loss‐of‐function evidence revealed that CPK5/CPK6 are essential for the accumulation of 4MI3G and camalexin by coordinately regulating the expression of genes in the tryptophan metabolism pathway in response to *B. cinerea* (Figures 4 and 5). Besides biosynthesis, the transportation of these active defense chemicals, 4M13G derivatives and camalexin, to the apoplastic space is also important for plants in resistance to *B. cinerea*. In *Arabidopsis*, the plasma membrane‐associated PEN3 is an important ATP binding cassette transporter of 4MI3G derivatives and camalexin (Lu et al. 2015; Xu et al. 2016; He et al. 2019). Recently, the role of PEN3 and its close homolog PDR12 serve as two major transporters mediating camalexin secretion for resistance to *B. cinerea* is identified (He et al. 2019). We found that the expression of *PDR12* and *PEN3* was greatly up‐regulated after CPK5 activation as well (Figure S5A). In response to *B. cinerea* infection, expression of *PDR12* and *PEN3* were dramatically and slightly compromised in *cpk5 cpk6* double mutant, respectively (Figure S5B), suggesting that CPK5/CPK6 are also involved in regulating the secretion of 4MI3G derivatives and camalexin in addition to their biosynthesis. It was reported that the sequential action of indole glucosinolates and camalexin is required in disease resistance to oomycete pathogen *Phytophthora brassicae*, a hemibiotrophic pathogen (Schlaeppi et al. 2010). We observed significantly compromised resistance of *cpk5 cpk6* double mutant to *B. cinerea* (Figure 1), which could, at least partially, result from the dramatic (~50%) reduction of 4MI3G and camalexin in the double mutant (Figure 4). Coordinated induction of the biosynthesis and secretion of camalexin and indole glucosinolates after activation of CPK5 could contribute greatly to plant resistance to *B. cinerea*.

Activation of CPKs and MAPK cascades are two of the earliest events during plant‐pathogen interaction (Hake and Romeis 2019). Both of them are involved in the regulation of biosynthesis and secretion of IGSs and camalexin in defense to *B. cinerea* (Ren et al. 2008; Xu et al. 2016; He et al. 2019; this report). Transcriptional expression pattern of genes in tryptophan metabolism pathway after activation of CPK5 is very similar to that after the activation of MPK3/MPK6 (Figure 2). However, we found that these two pathways are independent of each other. Gain‐of‐function activation of either CPK5/CPK6 or MPK3/MPK6 pathway is sufficient to induce the accumulation of 4MI3G and camalexin in the absence of the other (Figure 9), suggesting that they are independent of each other. In addition, both pathways are required for the full induction of 4MI3G and camalexin and an effective resistance against *B. cinerea* infection (Figure 7). Conditional *cpk5 cpk6 mpk3 mpk6* quadruple mutant plants showed a higher susceptibility to *B. cinerea* and significantly reduced accumulation of 4MI3G or camalexin than either *cpk5 cpk6* or conditional *mpk3 mpk6* double mutant in response to *B. cinerea* (Figure 7), suggesting that CPK5/CPK6 and MPK3/MPK6 signaling pathway could collaboratively regulate the biosynthesis of these important secondary metabolites and plant resistance in *B. cinerea*‐triggered immunity. It was reported that CPK5 activation regulates early‐responsive gene expression on the perception of PAMPs, which is either independently or coordinately with MAPK cascades (Boudsocq and Sheen 2013). Wounding‐induced ethylene biosynthesis mediated by CPK5/CPK6 is independent of the MPK3/MPK6 pathway as well (Li et al. 2018). These findings suggest that CPK5 and MPK3/MPK6 signaling function differentially but cooperatively to control several important defense responses in plant immunity.

Collectively, we propose an independent but cooperative interaction between CPK5/CPK6 and MPK3/MPK6 signaling in regulating camalexin and indole glucosinolates biosynthesis in response to *B. cinerea* infection (Figure 9E). The interplay between CPK5/CPK6 and MPK3/MPK6 signaling during this process could happen at several levels. Firstly, the secondary responses/signals after activation of one signaling pathway could result in the change of the activity of the other. For example, activation of CPK5 can induce ROS generation through directly phosphorylating RbohD (Dubiella et al. 2013). ROS can then signal additional defense responses. Such secondary responses may have an indirect effect on the activity of other signaling pathways, including the activation of MPK3/MPK6 cascade (Meng and Zhang 2013; Jalmi and Sinha 2015). Indeed, we detected phosphorylation activation of MPK3/MPK6 after CPK5 activation in *CPK5‐VK* transgenic plants (Figure S6), which is likely to be an indirect effect. It was shown that in the *cpk5 cpk6* mutant, the *B. cinerea*‐activated MPK3/MPK6 is comparable to that in the wild type (Figure 8), indicating that CPK5/CPK6 activity is not required for the activation of MPK3/MPK6 in plant defense against *B. cinerea*. Secondly, the interplay between CPK5/CPK6 and MPK3/MPK6 could converge on their common downstream targets to fine‐tune the specific defense response. Evidence revealed that MYB51 functions genetically downstream of both of CPK5/CPK6 and MPK3/MPK6 signaling pathways in regulating indole glucosinolates (Xu et al. 2016; Figure 6 in this report). At present, direct substrates of MPK3/MPK6 in regulating secondary metabolites biosynthesis have been identified. ERF6 regulates the metabolic flow of indole glucosinolates downstream of MPK3/MPK6. Phospho‐mimic form of ERF6 binds to the promoter of *CYP81F2, IGMT1*, and IGMT2 to drive the biosynthesis of 4MI3G (Xu et al. 2016). WRKY33 is required for MPK3/MPK6 mediated camalexin biosynthesis via directly binding to the promoter of *PAD3*, a key biosynthetic gene of camalexin (Mao et al. 2011). Other than *PAD3*, it was recently reported that WRKY33 directly binds to many genes in tryptophan‐derived metabolites pathway in plant immunity signaling (Birkenbihl et al. 2017), indicating its potential role in regulating both camalexin and indole glucosinolates biosynthesis. Our RNA‐seq and RT‐qPCR analyses revealed that *WRKY33* was highly up‐regulated after CPK5 activation (Figures 2A, S7), similar to that after MPK3/MPK6 activation. While we were preparing our manuscript, it was reported that WRKY33 can be phosphorylated by CPK5 in its DNA‐binding domain to enhance the DNA‐binding activity of WRKY33, which promotes the expression of biosynthetic genes in camalexin pathway (Zhou et al. 2020). Whether WRKY33 is also involved in the CPK5/CPK6‐regulated 4MI3G biosynthesis is currently unclear. Additional research is needed to further define the interplay between CPK5/CPK6 and MPK3/MPK6 pathways in signaling the multilayered immune responses in plants.

## MATERIALS AND METHODS

### Materials and growth condition

*Arabidopsis* Col‐0 ecotype is used as wild type control and the background of mutants and transgenic lines. *DD* (*GVG‐NtMEK2* ^*DD*^), *MPK6SR* (*mpk3 mpk6 P*_*MPK6*_:*MPK6* ^*YG*^, Line #58), *mkk4 mkk5* were reported in previous literatures of our laboratory (Ren et al. 2002; Xu et al. 2014; Li et al. 2018). T‐DNA insertion mutants *cpk5* (SAIL_657C06), *cpk6* (SALK_025460) and *cpk5 cpk6* double mutant were previously described (Gao et al. 2013; Li et al. 2018). *CPK5‐VK* and *CPK5‐FL* plants were generated by transforming GVG‐driven *CPK5‐VK* or *CPK5‐FL* into Col‐0, which could be induced by DEX. *CPK5‐VK* only contains variable domain(V) and kinase domain(K) of CPK5, with C‐terminal deleted, whereas *CPK5‐FL* contains the intact sequence.

Surface‐sterilized seeds were vernalized at 4°C for 2–4 d, then sown in Petri dishes containing 1/2 MS (Murashige and Skoog) liquid medium under 70 μmol m^−2^ s^−1^ continuous light at 22^o^C. After 6 d, the seedlings were transferred to 20‐mL gas chromatography (GC) vials with 6 mL liquid medium. Twelve‐d‐old seedlings were used for experiments. For soil‐growth plants, plants were grown in a growth chamber at 22 °C under 10‐h‐light/14‐h‐dark cycle with 100 μmol m^−2^ s^−1^ light intensity.

### *B. cinerea* **inoculation and chemical treatment**

For the *B. cinerea* resistance assay, the same position leaves of 4‐week‐old plants were detached and the petioles were inserted into the medium containing 0.8% plant agar or put on wet filter paper with 10 μM NA‐PP1 or DMSO. Then inoculate the leaves with 10 μL drops of *B. cinerea* spore suspension (5 × 10^5^ spores/mL). The lesion size was measured by ImageJ at indicated time points.

For the IGS and camalexin analysis, seedlings growing in 6 mL liquid medium were treated with *B. cinerea* (4 × 10^5^ spores per GC vial) or DEX (5 μM final concentration).

### Real‐time PCR analysis and RNA‐seq

After total RNA was extracted by TRIzol reagent (Invitrogen) and treated with DNase, 500 ng RNA was used for reverse transcription. A RealPlex^2^ real‐time PCR machine (Eppendorf) was used to perform the qPCR analysis. Gene expression levels were calculated as percentages of the *EF1α* transcript.

Two sets of independent repeated RNA samples were selected for profiling, which was performed by the Shenzhen BGI co. Ltd. (China). Briefly, RNA sequencing libraries were constructed using TruSeq RNA library preparation kit and sequenced using the Illumina HiSeq platform. After filtering out the reads with poor qualities using SOAPnuke software, clean reads were mapped to the *Arabidopsis* reference genome using HISAT and to reference gene sequences with Bowtie2. Gene expression levels were calculated with RSEM and shown as fragments per kb per million reads (FPKM) values. Differentially expressed genes (DEGs) were detected with NOIseq. Heatmap for analysis of DEGs was created in R. The clean reads generated from RNA‐seq analyses were deposited at NCBI Sequence Read Archive (PRJNA625276).

Gene ontology (GO) analysis was performed using the singular enrichment analysis (SEA) of AgriGo v2.0 with the *Arabidopsis* genome (TAIR10_2017) as the background input (Lee et al. 2018); GO terms significantly enriched were presented in Data Set S1 (false discovery rate <0.05). Then the GO results were combined into superclusters according to semantic similarity and visualized as treemap using REVIGO (Supek et al. 2011).

### Protein extraction and western blot

Seedling samples were collected at indicated times. Protein was extracted as previously described (Liu and Zhang 2004). The concentration of protein extracts was determined by the Bradford method. Anti‐HA (Sigma) antibody was used to detect CPK5‐VK protein expression in the immunoblot analyses. MAPK phosphorylation was detected using an anti‐pTEpY (Cell Signalling Technology) antibody (Xu et al. 2014).

### HPLC assay of glucosinolates

Quantitative analyses of GSs were performed as previously described (Miao et al. 2013; Xu et al. 2016). Briefly, freshly collected seedlings were boiled twice in 1 mL water for 10 min to extract GSs. Then the aqueous extract was applied to a DEAE‐Sephadex A‐25 column (30 mg, pyridine acetate form, Sigma) and treated by aryl sulphatase (Sigma) overnight. The desulphoglucosinolates were analyzed by an Angient 1260 Infinity II LC System equipped with a Spherisorb C18 column (5‐µm particle size, 4.6 mm × 250 mm). ONPG (Solarbio) was used as an internal standard.

### Measurement of camalexin

After treatment of seedlings in liquid medium, 1 mL culture solution was harvested at the corresponding time point, and 200 µL were added to the 96‐well opaque microplate. For regular measurements, values of fluorescence were measured with excitation light at 315 nm and emission light at 385 nm. For the medium pretreated with NA‐PP1 or DMSO, the excitation light was changed to 334 nm to minimize the interference of NA‐PP1. The standard curve was made with camalexin standard samples.

### Statistical analyses

GraphPad Prism was used for statistical analysis of the data. One‐way ANOVA followed by Tukey's post hoc test was conducted to evaluate the statistical significance among different genotypes at a single time point. One‐way ANOVA followed by Dunnett's multiple comparison test was conducted to evaluate the statistical significance between each mutant and Col‐0 at a single time point. Two‐way ANOVA was performed to determine whether two groups of data were significantly different. Different lowercase letters are used to indicate differences that are statistically significant. The experiments were repeated independently at least twice with similar results.

### Accession numbers

Sequence data used in this article can be found in The *Arabidopsis* Information Resource (TAIR) or Genbank database under the following accession numbers: *CPK5* (At4g35310), *CPK6* (At2g17290), *ASA* (AT5G05730), *ASB* (AT1G25220), *PAT* (AT5G17990), *IGPS* (At2g04400), *TSA* (AT3G54640), *TSB* (AT5G54810), *CYP79B2* (At4g39950), *CYP79B3* (At2g22330), *CYP81F2* (AT5G57220), *IGMT1* (At1g21100), *IGMT2* (At1g21120), *CYP71A13* (AT2G30770), *CYP71A12* (AT2G30750), *PAD3* (AT3G26830), *CYP83B1* (AT4G31500), *UGT74B1* (AT1G24100), *ST5a* (AT1G74100), *PDR12* (AT1G15520), *PEN3* (At1G59870), *MYB51* (At1g18570), *MYB122* (At1g74080), and *EF1α* (At5g60390).

## AUTHOR CONTRIBUTIONS

L.Y., J.X., and S.Z. designed the project. L.Y., Y.Z., R.G., S.L., and X.X. performed the experiments. L.Y., S.Z., and J.X. analyzed the results and wrote the manuscript. All authors read and approved the manuscript.

## COMPETING INTERESTS

The authors declare no competing financial interest.

## Supporting information

Additional Supporting Information may be found online in the supporting information tab for this article: http://onlinelibrary.wiley.com/doi/10.1111/jipb.12973/suppinfo

**Figure S1**. Gene ontology (GO) enrichment analysis of differentially expressed genes after activation of CPK5 and MPK3/MPK6
(**A**) Venn diagram represents the numbers of differentially expressed genes (DEGs) in *DD* and *CPK5‐VK* (DEX treatment 6 h compared to 0 h, probability>0.9, fold>2). Red “↑” represents numbers of up‐regulated genes; Blue “↓” represents numbers of down‐regulated genes; numbers in black represents total DEGs. (**B**) Hierarchical clustering analysis of *DD* and *CPK5‐VK* DEGs. Color key represents the log_2_(ratios) between DEX treatment 6 h and 0 h. (**C**) Gene ontology enrichment analysis. Treemap view of REVIGO for biological process on CPK5 and MPK3/MPK6 both up‐regulated genes. Each rectangle is a GO term enriched by agriGO, then the results are combined into superclusters according to semantic similarity and visualized with different colors using REVIGO. Rectangle size is adjusted to reflect the abs log_10_(*P*‐value) of the GO term in the underlying Gene Ontology Annotation database.
**Figure S2**. Expression of genes in IAOx biosynthesis pathway is compromised in *cpk5 cpk6* double mutant
Twelve‐d‐old Col‐0, *cpk5 cpk6, cpk5*, and *cpk6* plants grown in liquid medium were treated with *B. cinerea* spores (4 × 10^5^ spores/mL) for indicated times. Expression levels of IAOx biosynthesis genes were determined by RT‐qPCR and calculated as percentages of the *EF1α* transcript. Values are means ± *SD*, n = 3. One‐way ANOVA was performed to compare gene expression level of between mutants and Col‐0 at 12 h and 18 h, *P < 0.05, **P < 0.01, ***P < 0.001.
**Figure S3**. Loss of function of CPK5/CPK6 results in no change in I3G accumulation in response to *B. cinerea* infection
Twelve‐d‐old Col‐0, *cpk5 cpk6, cpk5*, and *cpk6* plants grown in liquid medium were treated with *B. cinerea* spores (4 × 10^5^ spores/mL). Levels of I3G were measured at indicated time points. Values are means ± *SD*, n = 3. Two‐way ANOVA analysis revealed that no significant difference exists in I3G level change among different genotypes.
**Figure S4**. *B. cinerea*‐induced expression of I3G biosynthetic genes is not compromised in *cpk5 cpk6* double mutants
Twelve‐d‐old Col‐0, *cpk5 cpk6, cpk5*, and *cpk6* plants grown in liquid medium were treated with *B. cinerea* spores (4 × 10^5^ spores/mL) for indicated times. Gene expression was determined by RT‐qPCR and calculated as percentages of the *EF1α* transcript. Values are means ± *SD*, n = 3. One‐way ANOVA was performed to compare gene expression in mutants and Col‐0 at 6 h, 12 h, and 18 h. *P < 0.05, **P < 0.01, ***P < 0.001.
**Figure S5**. Expression of camalexin transporter *PDR12* and *PEN3* is regulated by CPK5/CPK6
(**A**) Twelve‐d‐old *CPK5‐VK* plants grown in liquid medium were treated with 5 μM DEX for indicated times. Transcript levels were quantified by RT‐qPCR and calculated as percentages of the *EF1α* transcript. Values are means ± *SD*, n = 3. (**B**) Twelve‐d‐old Col‐0, *cpk5 cpk6, cpk5*, and *cpk6* plants grown in liquid medium were treated with *B. cinerea* spores (4 × 10^5^ spores/mL) for indicated times. Transcript levels were quantified by RT‐qPCR and calculated as percentages of the *EF1α* transcript. Values are means ± *SD, n* = 3. One‐way ANOVA was performed to compare the gene expression level in mutants and Col‐0 at 12 h and 18 h, *P < 0.05, **P < 0.01, ***P < 0.001.
**Figure S6**. MPK3/MPK6 is activated in *CPK5‐VK* plants after DEX treatment
Protein levels of CPK5‐VK in *CPK5‐VK* plants after DEX treatment were determined by immunoblot analysis using an anti‐HA antibody (top panel). Activation of MPK3/MPK6 in *CPK5‐VK* plants after DEX treatment was determined by immunoblot analysis using an anti‐pTEpY antibody (middle panel). Equal loading of proteins was confirmed by Coomassie brilliant blue staining (bottom panel).
**Figure S7**. Expression of *WRKY33* is highly induced after activation of CPK5 in DEX‐treated *CPK5‐VK* plants
Twelve‐d‐old *CPK5‐VK* plants grown in liquid medium were treated with 5 μM DEX for indicated times. Transcript levels were quantified by RT‐qPCR and calculated as percentages of the *EF1α* transcript. Values are means ± *SD, n* = 3.
**Table S1**. The primers for RT‐qPCR analyses in this study.

**Data Set S1**. Differentially expressed genes shared in *CPK5‐VK* and *DD* transgenic plants after DEX treatment and GO analysis data.
