# Supplementary material for: Co‐regulation of indole glucosinolates and camalexin biosynthesis by CPK5/CPK6 and MPK3/MPK6 signaling pathways
Source: J Integr Plant Biol. 2020 Jun 26;62(11):1780–96. doi: 10.1111/jipb.12973 (PMC7687085; doi:10.1111/jipb.12973)
Supplement: Supplementary file 1 — Figure S1. Gene ontology (GO) enrichment analysis of differentially expressed genes after activation of CPK5 and MPK3/MPK6 (A) Venn diagram represents the numbers of differentially expressed genes (DEGs) in DD and CPK5‐VK (DEX treatment 6 h compared to 0 h, probability>0.9, fold>2). Red “↑” represents numbers of up‐regulated genes; Blue “↓” represents numbers of down‐regulated genes; numbers in black represents total DEGs. (B) Hierarchical clustering analysis of DD and CPK5‐VK DEGs. Color key represents the log2(ratios) between DEX treatment 6 h and 0 h. (C) Gene ontology enrichment analysis. Treemap view of REVIGO for biological process on CPK5 and MPK3/MPK6 both up‐regulated genes. Each rectangle is a GO term enriched by agriGO, then the results are combined into superclusters according to semantic similarity and visualized with different colors using REVIGO. Rectangle size is adjusted to reflect the abs log10(P‐value) of the GO term in the underlying Gene Ontology Annotation database. Figure S2. Expression of genes in IAOx biosynthesis pathway is compromised in cpk5 cpk6 double mutant Twelve‐d‐old Col‐0, cpk5 cpk6, cpk5, and cpk6 plants grown in liquid medium were treated with B. cinerea spores (4 × 10^5^ spores/mL) for indicated times. Expression levels of IAOx biosynthesis genes were determined by RT‐qPCR and calculated as percentages of the EF1α transcript. Values are means ± SD, n = 3. One‐way ANOVA was performed to compare gene expression level of between mutants and Col‐0 at 12 h and 18 h, *P < 0.05, **P < 0.01, ***P < 0.001. Figure S3. Loss of function of CPK5/CPK6 results in no change in I3G accumulation in response to B. cinerea infection Twelve‐d‐old Col‐0, cpk5 cpk6, cpk5, and cpk6 plants grown in liquid medium were treated with B. cinerea spores (4 × 10^5^ spores/mL). Levels of I3G were measured at indicated time points. Values are means ± SD, n = 3. Two‐way ANOVA analysis revealed that no significant difference exists in I3G level change among different [file JIPB-62-1780-s001.docx]

**Supplementary Information**


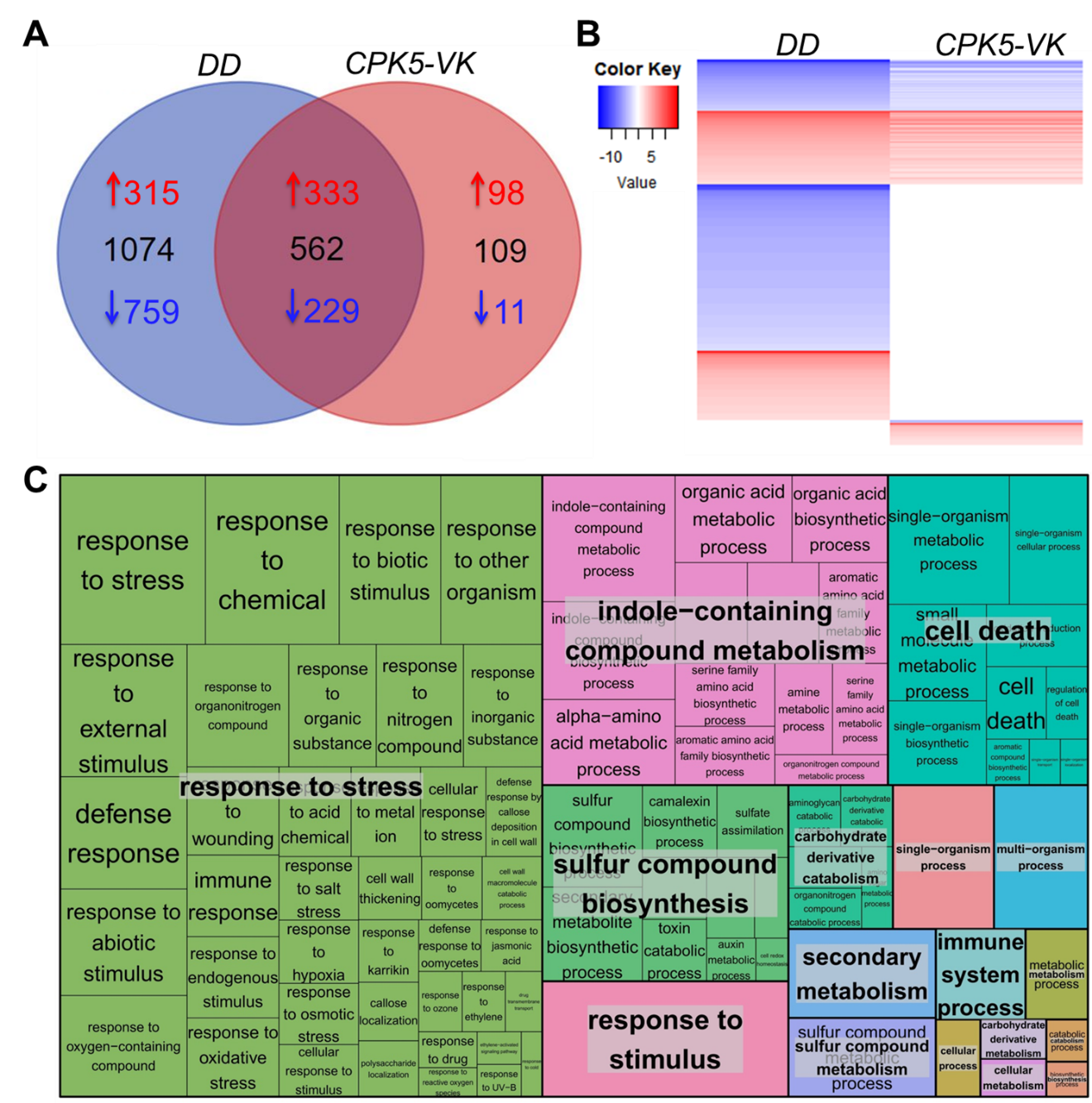


**Figure S1. Gene ontology (GO) enrichment analysis of differentially expressed genes after activation of CPK5 and MPK3/MPK6.**

(A) Venn diagram represents the numbers of differentially expressed genes (DEGs) in *DD* and *CPK5-VK* (DEX treatment 6 h compared to 0 h, probability > 0.9, fold > 2). Red “↑” represents numbers of up-regulated genes; Blue “↓” represents numbers of down-regulated genes; numbers in black represents total DEGs. (B) Hierarchical clustering analysis of *DD* and *CPK5-VK* DEGs. Color key represents the log_2_(ratios) between DEX treatment 6 h and 0 h.

(C) Gene ontology enrichment analysis. Treemap view of REVIGO for biological process on CPK5 and MPK3/MPK6 both up-regulated genes. Each rectangle is a GO term enriched by agriGO, then the results are combined into superclusters according to semantic similarity and visualized with different colors using REVIGO. Rectangle size is adjusted to reflect the abs log_10_(P-value) of the GO term in the underlying Gene Ontology Annotation database.


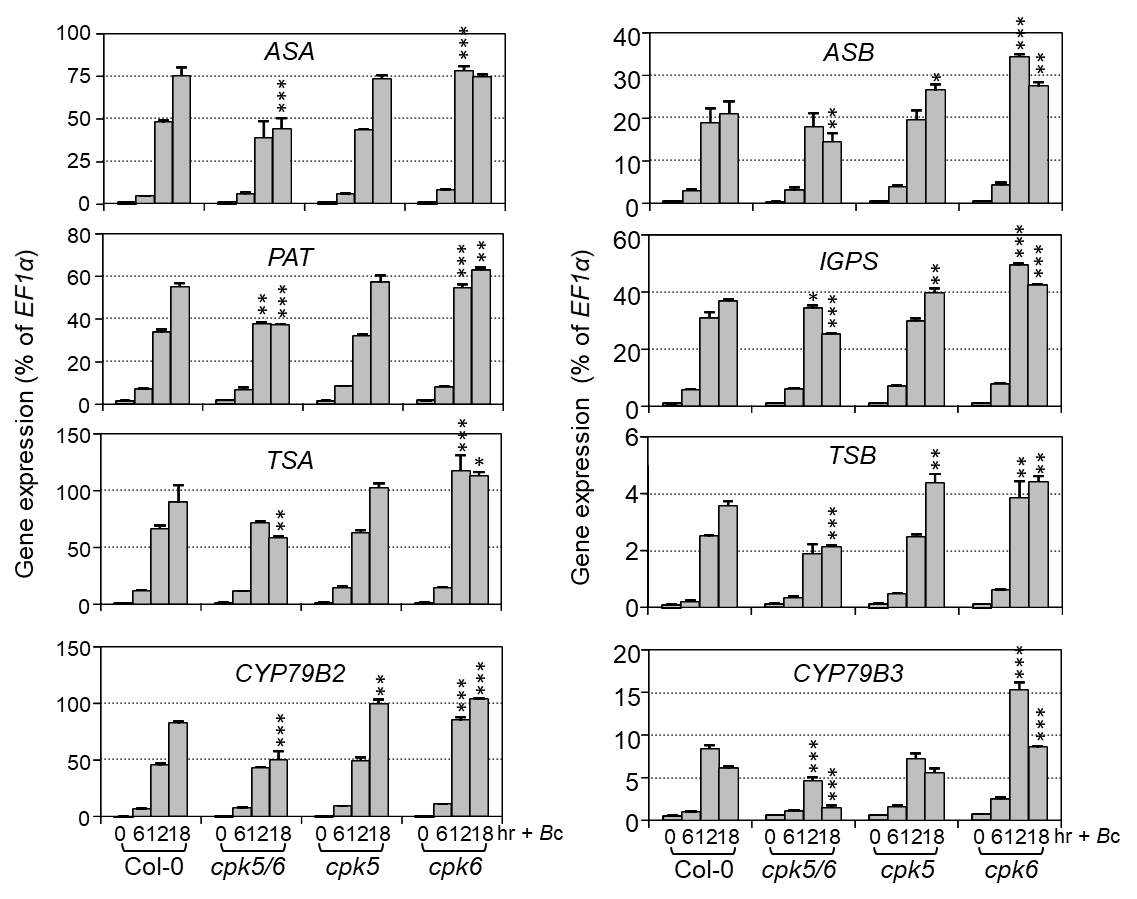


**Figure S2. Expression of genes in IAOx biosynthesis pathway is compromised in *cpk5 cpk6* double mutant**

Twelve-day-old Col-0, *cpk5 cpk6*, *cpk5*, and *cpk6* plants grown in liquid medium were treated with *B. cinerea* spores (4×10^5^ spores/mL) for indicated times. Expression levels of IAOx biosynthesis genes were determined by RT-qPCR and calculated as percentages of the *EF1α* transcript. Values are means ± SD, n = 3. One-way ANOVA was performed to compare gene expression level of between mutants and Col-0 at 12h and 18 h, *P < 0.05，**P < 0.01, ***P < 0.001.


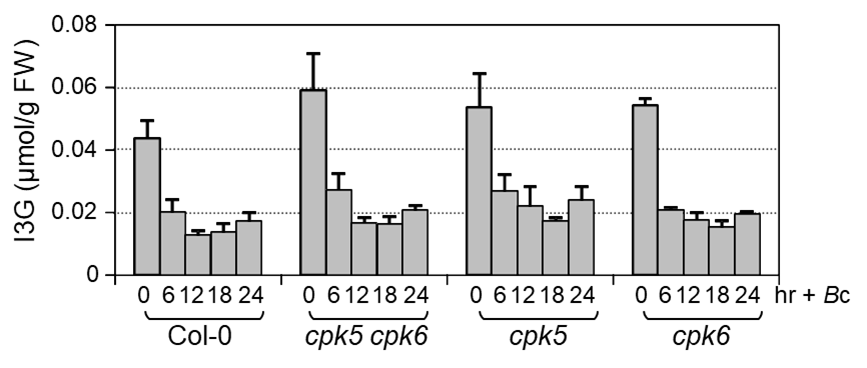


**Figure S3. Loss of function of CPK5/CPK6 results in no change in I3G accumulation in response to *B. cinerea* infection.**

Twelve-day-old Col-0, *cpk5 cpk6*, *cpk5*, and *cpk6* plants grown in liquid medium were treated with *B. cinerea* spores (4×10^5^ spores/mL). Levels of I3G were measured at indicated time points. Values are means ± SD, *n* = 3. Two-way ANOVA analysis revealed that no significant difference exists in I3G level change among different genotypes.


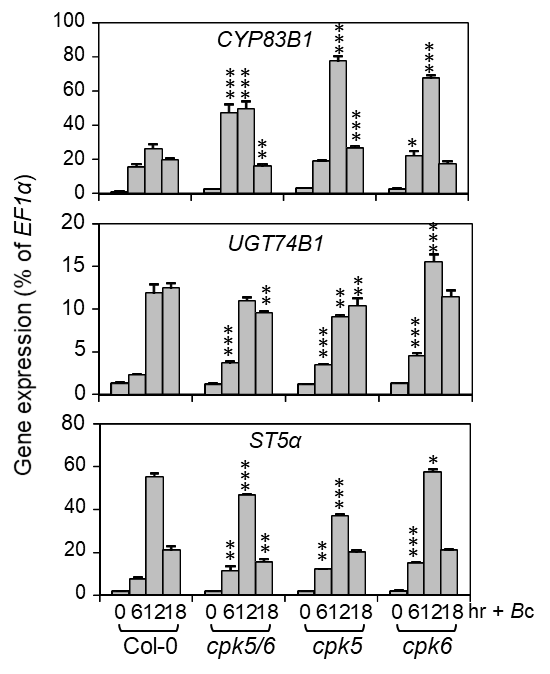


**Figure S4. *B. cinerea*-induced expression of I3G biosynthetic genes is not compromised in *cpk5 cpk6* double mutants.**

Twelve-day-old Col-0, *cpk5 cpk6*, *cpk5*, and *cpk6* plants grown in liquid medium were treated with *B. cinerea* spores (4×10^5^ spores/mL) for indicated times. Gene expression was determined by RT-qPCR and calculated as percentages of the *EF1α* transcript. Values are means ± SD, *n* = 3. One-way ANOVA was performed to compare gene expression in mutants and Col-0 at 6 h, 12 h and 18 h. *P < 0.05，**P < 0.01, ***P < 0.001.


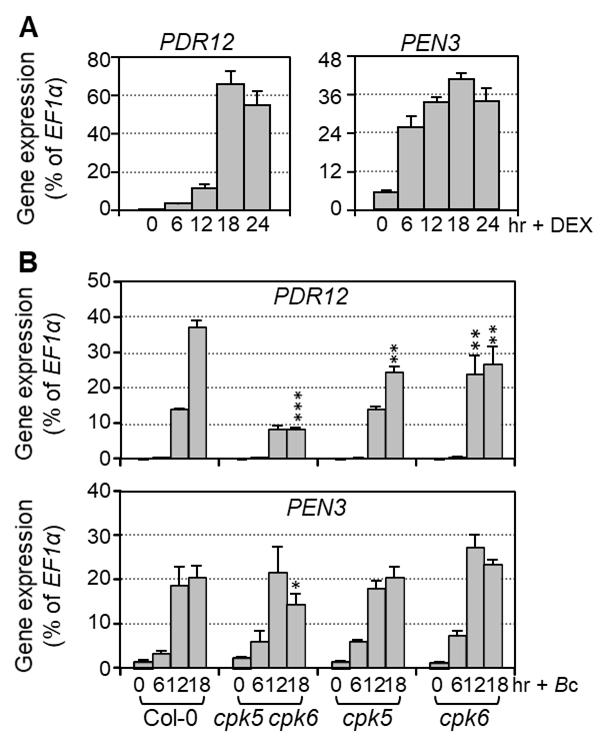


**Figure S5. Expression of camalexin transporter *PDR12* and *PEN3* is regulated by CPK5/CPK6.**

(A) Twelve-day-old *CPK5-VK* plants grown in liquid medium were treated with 5 μM DEX for indicated times. Transcript levels were quantified by RT-qPCR and calculated as percentages of the *EF1α* transcript. Values are means ± SD, n = 3. (B) Twelve-day-old Col-0, *cpk5 cpk6*, *cpk5*, and *cpk6* plants grown in liquid medium were treated with *B. cinerea* spores (4×10^5^ spores/mL) for indicated times. Transcript levels were quantified by RT-qPCR and calculated as percentages of the *EF1α* transcript. Values are means ± SD, n = 3. One-way ANOVA was performed to compare the gene expression level in mutants and Col-0 at 12h and 18 h, *P < 0.05，**P < 0.01, ***P < 0.001.


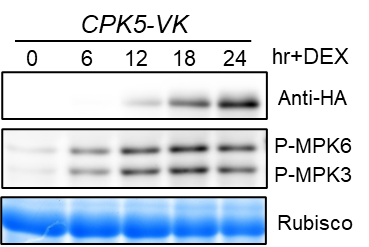


**Figure S6. MPK3/MPK6 is activated in *CPK5-VK* plants after DEX treatment.**

Protein levels of CPK5-VK in *CPK5-VK* plants after DEX treatment were determined by immunoblot analysis using an anti-HA antibody (top panel). Activation of MPK3/MPK6 in *CPK5-VK* plants after DEX treatment was determined by immunoblot analysis using an anti-pTEpY antibody (middle panel). Equal loading of proteins was confirmed by Coomassie brilliant blue staining (bottom panel).


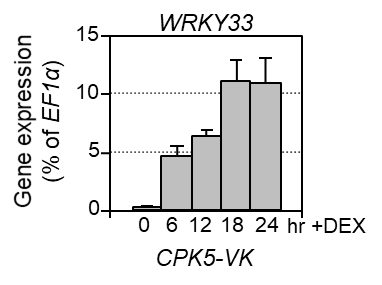


**Figure S7. Expression of *WRKY33* is highly induced after activation of CPK5 in DEX-treated *CPK5-VK* plants.**

Twelve-day-old *CPK5-VK* plants grown in liquid medium were treated with 5 μM DEX for indicated times. Transcript levels were quantified by RT-qPCR and calculated as percentages of the *EF1α* transcript. Values are means ± SD, *n* = 3.

**Table S1: Primer pairs used for RT-qPCR**

| **Primer pairs used for RT-qPCR** | | |
| --- | --- | --- |
| **Gene** | **Forward (5'-3')** | **Backward (5'-3')** |
| *ASA* | GCAACGATGTTGGAAAGGTT | ATTCTCCTGTCACCGTGGAG |
| *ASB* | TCTGGGATTTCCTTGCAAAC | GTGACCGCACAATCTTTCCT |
| *PAT* | CGAGGTGGAGGTCCAGACTA | CGGTTGCTAACCAGAAGAGC |
| *IGPS* | GTTGGCGAATCTGGTCTGT | TTCTCAGGGTCGTTCTGCTT |
| *TSA* | GTTCCCGATGTTCCTCTTGA | CTCTGTTGGTGTGGTTGGTG |
| *TSB* | AACAAGCGATGGAGAAATGG | TTCGAACCTCTGTGTCATCG |
| *CYP79B2* | GCCGGATATCACATCCCTAA | TCCGGTTTAAAGCAAAGTGG |
| *CYP79B3* | CGTGGCACTCTCTGATACGA | CAGACCAAACCTTGGGGTTA |
| *CYP71A13* | GGGTAGAGGCTGGACCAAAT | ACAACCGAAGATGGAAATGC |
| *CYP71A12* | GGATATGTTTATAGGAGGG | CTTGGAGTTTCTTCATAACA |
| *CYP71B15* (*PAD3*) | GGTACGGGATAAATCTCTATGA | AGATACAGTCGATGAACCTAC |
| *CYP81F2* | CAGCTGCACCACTTCTTGTTC | AGGCATAAACTTCTCGGGCTC |
| *IGMT1* | AAGTGTGGTAAGGCCTTATCC | ACCACATCTTTCAACTGTGCC |
| *IGMT2* | CAAGTGTGAGAAGGTCTCCGTA | ACCACGTCTTTCAGTTGTGC |
| *MYB51* | TCAACGAGTTCTTCCTTCGCA | ACGGAGGAATCAGAGAACGTG |
| *MYB122* | CAGGATCATCATCAGCTCGGT | CGTTGACCTCTCACCTTCTGA |
| *CYP83B1* (*SUR2*) | ATTCCGGCCAAGACCATCATT | GCCCTTGAAGTCCACTCCTTT |
| *UGT74B1* | TGGAAGGGTTGAGTTTGGGAG | ACGATTACTTCCCCAGCTTCC |
| *ST5α* | TGCTGAGAAAGTGGTGAAGCT | AATACGAGCAGCCATCTCAGG |
| *PDR12* | TATGACGCCTAACCACCACA | TGGGCAAAGCCAGTAGTACC |
| *PEN3* | GATGCTCGAAGCTAGCTCACTC | CTTTTACCAACGCTTTGTTTCG |
| *WRKY33* | GTGATATTGACATTCTTGACGA | GATGGTTGTGCACTTGTAGTA |
